# Supplementary material for: Perceived barriers to care for migrant children and young people with mental health problems and/or neurodevelopmental differences in high-income countries: a meta-ethnography
Source: BMJ Open. 2021 Sep 16;11(9):e045923. doi: 10.1136/bmjopen-2020-045923 (PMC8449985; doi:10.1136/bmjopen-2020-045923)
Supplement: Supplementary data [file bmjopen-2020-045923supp001.pdf]

exp Mental Health Services/ or Psychiatric Nursing/ or ((mental or psycholog* or psychiat*) adj3 (care or counseling or nursing or service* or treatment)).ti,ab,kf.

Culturally Competent Care/ or exp Attitude of Health Personnel/ or Health Equity/ or exp "Health Services Needs and Demand"/ or Medically Underserved Area/ or exp Health Resources/ or Delivery of Health Care/ or exp Patient Acceptance of Health Care/ or exp Attitude to Health/ or Health Services Accessibility/ or Healthcare Disparities/ or ((access* or accept* or adherence or attitude* or barrier* or compliance or deliver* or disparit* or engag* or facilitat* or hinder* or hindrance* or impede* or inequalit* or involve* or nonaccept* or non-accept or non-show* or obstacle* or participat* or refus* or seek* or usage* or utili*) adj3 (care or counseling or healthcare or health care or health-care or nursing or service* or treat*)).ti,ab,kf.

(exp Emigrants/ and Immigrants/) or Refugees/ or "Transients and Migrants"/ or "Emigration and Immigration"/ or (asylum seek* or asylum-seeker* or emigrant* or immigrant* or migrant* or refugee* or transient* or foreign born).ti,ab,kf.

(child**minor****).ti,ab,kf.
