# Supplementary material for: Perceived barriers to care for migrant children and young people with mental health problems and/or neurodevelopmental differences in high-income countries: a meta-ethnography
Source: BMJ Open. 2021 Sep 16;11(9):e045923. doi: 10.1136/bmjopen-2020-045923 (PMC8449985; doi:10.1136/bmjopen-2020-045923)
Supplement: Supplementary data [file bmjopen-2020-045923supp002.pdf]

Supplementary Table 1. Development of third-order constructs from second-order constructs: a worked example.

Study	Second-order construct (themes from included studies)	Third-order construct (new theme)
Bradby et al., 2007 [1]	Discriminatory health services	Service providers lack cultural responsiveness
Islam et al., 2017 [2]	Lack of South Asian representation in mental health care and promotion	
Posselt et al., 2017 [3]	• Lack of funding for improving the cultural responsiveness of services<br>• Services perceived as discriminatory or not culturally appropriate	
Wang et al., 2018 [4]	Providers lack cultural competence and sensitivity	
McCann et al., 2016 [5]	Perceived lack of cultural competency of formal help sources	
Choummanivong, 2013 [6]	Different understandings of an issue	
Jacobs, 2014 [7]	• Trust: lack of Somali mental health care providers<br>• Lack of provider education	
Smith, 2017 [8]	[Lack of] ethnically similar mentors	

REFERENCES

1 Bradby H, Varyani M, Oglethorpe R, *et al.* British Asian families and the use of child and adolescent mental health services: A qualitative study of a hard to reach group. *Soc Sci Med* 2007;**65**:2413–24. doi:10.1016/j.socscimed.2007.07.025

2 Islam F, Multani A, Hynie M, *et al.* Mental health of South Asian youth in Peel Region, Toronto, Canada: A qualitative study of determinants, coping strategies and service access. *BMJ Open* 2017;**7**:e018265. doi:10.1136/bmjopen-2017-018265

3 Posselt M, McDonald K, Procter N, *et al.* Improving the provision of services to young people from refugee backgrounds with comorbid mental health and substance use problems: Addressing the barriers. *BMC Public Health* 2017;**17**:280. doi:10.1186/s12889-017-4186-y

4 Wang C, Do KA, Frese K, *et al.* Asian Immigrant Parents' Perception of Barriers Preventing Adolescents from Seeking School-Based Mental Health Services. *School Ment Health* 2019;**11**:364–77. doi:10.1007/s12310-018-9285-0

5 McCann T V, Mugavin J, Renzaho A, *et al.* Sub-Saharan African migrant youths' help-seeking barriers and facilitators for mental health and substance use problems: A qualitative study. *BMC Psychiatry* 2016;**16**:275. doi:10.1186/s12888-016-0984-5

6 Choummanivong C. *Refugee Youths: Adaptation and Mental Health Service Provision*. 2013.

7 Jacobs M. *Better Mental Health Service Provision for Somali Youth: Overcoming the Barriers*. 2014.

8 Smith LT. 'They might not show it and they might not say it, but in their mind they're really stressed' : a qualitative exploration of stressors and barriers to care for Latinx youth. Published Online First: 2017.https://repositories.lib.utexas.edu/bitstream/handle/2152/47450/SMITH-MASTERSREPORT-2017.pdf
