# Supplementary material for: Perceived barriers to care for migrant children and young people with mental health problems and/or neurodevelopmental differences in high-income countries: a meta-ethnography
Source: BMJ Open. 2021 Sep 16;11(9):e045923. doi: 10.1136/bmjopen-2020-045923 (PMC8449985; doi:10.1136/bmjopen-2020-045923)
Supplement: Author's manuscript [file bmjopen-2020-045923.draft_revisions.pdf]

BMJ Open is committed to open peer review. As part of this commitment we make the peer review history of every article we publish publicly available.

When an article is published we post the peer reviewers' comments and the authors' responses online. We also post the versions of the paper that were used during peer review. These are the versions that the peer review comments apply to.

The versions of the paper that follow are the versions that were submitted during the peer review process. They are not the versions of record or the final published versions. They should not be cited or distributed as the published version of this manuscript.

BMJ Open is an open access journal and the full, final, typeset and author-corrected version of record of the manuscript is available on our site with no access controls, subscription charges or pay-per-view fees (http://bmjopen.bmj.com).

If you have any questions on BMJ Open's open peer review process please email info.bmjopen@bmj.com

BMJ Open

Perceived barriers to care for migrant children and young people with mental and neurodevelopmental health problems in high-income countries: a meta-ethnography.

Journal:	*BMJ Open*
Manuscript ID	bmjopen-2020-045923
Article Type:	Original research
Date Submitted by the Author:	18-Oct-2020

Complete List of Authors:	Place, Vanessa; Karolinska Institutet, Department of Public Health Sciences<br>Nabb, Benjamin; Karolinska Institutet, Department of Public Health Sciences<br>Gubi, Ester; Karolinska Institutet, Department of Public Health Sciences<br>Assel, Karima; Karolinska Institutet, Department of Public Health Sciences<br>Åhlén, Johan; Karolinska Institutet, Department of Public Health Sciences<br>Hagström, Ana; Karolinska Institutet, Department of Public Health Sciences<br>Bäärnhielm, Sofie; Transcultural Center<br>Dalman, Christina; Karolinska Institutet, Public Health Sciences<br>Hollander, Anna-Clara; Karolinska Institutet, Department of Public Health Sciences

Keywords:	PUBLIC HEALTH, Child & adolescent psychiatry < PSYCHIATRY, QUALITATIVE RESEARCH

SCHOLARONE™ Manuscripts

I, the Submitting Author has the right to grant and does grant on behalf of all authors of the Work (as defined in the below author licence), an exclusive licence and/or a non-exclusive licence for contributions from authors who are: i) UK Crown employees; ii) where BMJ has agreed a CC-BY licence shall apply, and/or iii) in accordance with the terms applicable for US Federal Government officers or employees acting as part of their official duties; on a worldwide, perpetual, irrevocable, royalty-free basis to BMJ Publishing Group Ltd ("BMJ") its licensees and where the relevant Journal is co-owned by BMJ to the co-owners of the Journal, to publish the Work in this journal and any other BMJ products and to exploit all rights, as set out in our licence.

The Submitting Author accepts and understands that any supply made under these terms is made by BMJ to the Submitting Author unless you are acting as an employee on behalf of your employer or a postgraduate student of an affiliated institution which is paying any applicable article publishing charge ("APC") for Open Access articles. Where the Submitting Author wishes to make the Work available on an Open Access basis (and intends to pay the relevant APC), the terms of reuse of such Open Access shall be governed by a Creative Commons licence – details of these licences and which Creative Commons licence will apply to this Work are set out in our licence referred to above.

Other than as permitted in any relevant BMJ Author's Self Archiving Policies, I confirm this Work has not been accepted for publication elsewhere, is not being considered for publication elsewhere and does not duplicate material already published. I confirm all authors consent to publication of this Work and authorise the granting of this licence.

For peer review only

Perceived barriers to care for migrant children and young people with mental and neurodevelopmental health problems in high-income countries: a meta-ethnography.

Vanessa Place[1], Benjamin Nabb[1], Ester Gubi[1], Karima Assel[1,2,3], Johan Åhlen[1,3], Ana Hagström[1,3], Sofie Bäärnhielm[2,4], Christina Dalman[1,3], Anna-Clara Hollander[1]

1. Karolinska Institutet, Department of Public Health Sciences, Stockholm, Sweden

2. Transkulturellt Centrum, Stockholm, Sweden

3. Centre for Epidemiology and Community Medicine, Stockholm, Sweden

4. Karolinska Institutet, Department of Clinical Neuroscience, Stockholm, Sweden

Corresponding author: Vanessa Place, vanessa.place@stud.ki.se , research group: Epidemiology of Psychiatric Conditions, Substance use and Social environment (EPICSS), Department of Public Health Sciences, Karolinska Institutet, Solnavägen 1E, SE-171 77, Stockholm, phone number: +46707567136

Main text word count -7077 words

For peer review only

ABSTRACT

Objectives – To develop conceptual understanding of perceived barriers to seeking care for migrant children and young people (aged 0-25 years) with mental and neurodevelopmental health problems in high-income countries.

Design – Qualitative evidence synthesis using meta-ethnography methodology. We searched four electronic databases (Medline; PsycINFO; Global Health; and Web of Science) from inception until July 2019 for qualitative studies exploring barriers to care (as perceived by migrant communities and service providers) for migrant children and young people in high-income countries with neurodevelopmental and mental health problems. The quality of included studies was explored systematically using a quality assessment tool.

Results – We screened 753 unique citations and 101 full texts, and 30 studies met our inclusion criteria. We developed twenty themes representing perceived barriers to care on the supply and demand sides of the care-seeking process. Barriers included: stigma; fear and mistrust of services; lack of information on mental health; and service providers with low cultural responsiveness. Themes were incorporated into Levesque and colleagues' conceptual framework of patient-centred access to healthcare, creating a version of the framework specific to migrant children and young people's mental and neurodevelopmental health.

Conclusions – This is the first qualitative evidence synthesis on barriers to care for mental and neurodevelopmental health problems in migrant children and young people in high-income countries. We present an adapted conceptual framework that will help professionals and policy makers to visualise the dynamic, complex nature of barriers to care, and assist in improving practice and designing interventions to overcome them. Similar barriers were identified across study participants and migrant populations. While many barriers were also similar to those for children and young people in general populations, migrant families faced further, specific barriers to care. Interventions targeting multiple barriers may be required to ensure migrant families reach care for these conditions.

Keywords – migrants, children and young people, mental health, neurodevelopmental disorders, care-seeking, qualitative research, meta-ethnography, qualitative evidence synthesis

ARTICLE SUMMARY

Strengths and limitations of this study

- To our knowledge, this is the first qualitative evidence synthesis of perceived barriers to care for migrant children and young people with mental and neurodevelopmental health problems in high-income countries.
- Meta-ethnography provides a systematic way of synthesising qualitative data across multiple studies.
- Meta-ethnography provides reviewers' interpretations of qualitative findings in a line-of-argument synthesis, creating a higher level of interpretation.
- The CASP (Critical Appraisal Skills Programme) quality assessment tool was used to explore the quality of included studies, but there is currently no consensus on how to assess the quality of primary qualitative studies.
- Qualitative research that explores the lived experiences of migrant communities and service providers can help shape future approaches to improving access to care.

For peer review only

INTRODUCTION

Over the last three decades, the numbers of migrants arriving in high-income countries (HICs) has risen substantially, an increasing proportion of whom are fleeing persecution and conflict [1]. The ongoing COVID-19 pandemic is expected to push millions more into poverty, driving further increases in international migration as borders reopen [2]. In 2019, the number of international migrants (people living in a country other than that in which they were born) reached 272 million, of which 33 million were children [3]. Moreover, the United Nations High Commissioner for Refugees (UNHCR) estimates that children make up approximately half of the 26 million refugees worldwide [4].

A body of evidence points to an increased risk of mental health problems amongst children and young people (aged up to 25 years) from migrant backgrounds, which does vary amongst this diverse population [5–9]. These diverse backgrounds and psychiatric healthcare needs present a unique challenge to healthcare systems. One study comparing the mental health of Swedish-born and immigrant adolescents found that immigrant adolescents reported more mental health problems than their peers, but long-term trends in mental health problems did not differ between the groups [9]. A systematic review on the mental health of refugee children in OECD countries found that refugee children seem to experience generally elevated levels of psychological distress, including symptoms of post-traumatic stress disorder (PTSD) and depression [10]. However, this review reported variation in the definition, measurement, and levels of psychological distress [10], demonstrating a need to better understand the mental health care needs of this diverse population.

Studies have demonstrated that psychiatric care usage is lower in migrant groups than general populations, an effect that reduces over time spent in the hosting country [11–14], and studies indicate that this under-utilisation is present among migrant children and young people in HICs [15,16]. The reasons behind this are still not understood. Thus, bringing together qualitative research exploring barriers to children and young people's mental health services is essential to form a comprehensive understanding of this phenomenon from the perspective of those who experience it.

The aim of this qualitative synthesis was to develop conceptual understanding of perceived barriers to care-seeking for children and young people (aged up to 25 years) from a migrant background, hereafter referred to as migrant children and young people, with mental health problems and neurodevelopmental disorders in HICs. The research question was: what are the barriers, as perceived by migrant communities and service providers, to seeking care for migrant children and young people with mental and neurodevelopmental health problems in HICs? The purpose of this synthesis was to inform the design of interventions to increase care-seeking from mental health services amongst newly-arrived migrant children and young people.

The initial focus of the synthesis was mental health problems, but preliminary literature searches revealed that the body of literature exploring barriers to care for children's neurodevelopmental disorders (including autism spectrum disorders, ASD, and attention deficit hyperactivity disorder, ADHD) was sometimes included within the mental health frame. As the context of these studies and perceived barriers were similar to those for mental health problems, and care for these conditions is often given within mental health care systems, the synthesis aim was modified and these studies included.

Our chosen methodology was Nobilt and Hare's meta-ethnography, an interpretive approach to qualitative synthesis that generates a higher level of interpretation or theory [17]. The synthesis results were used to adapt Levesque and colleagues' framework of patient-centred access to healthcare [18], presented in its original form in Figure 1. The framework defines access to healthcare is defined as "the opportunity to identify healthcare needs, to seek healthcare services, to reach, to obtain or use healthcare services, and to actually have a need for services fulfilled." Five dimensions of

For peer review only

accessibility, and five corresponding abilities of populations, interact to generate access (Figure 1) [18]. The results of our meta-ethnography provide a new interpretation and application of this framework specific to migrant children and young people's mental health.

METHODOLOGY

We followed Nobilt and Hare's methodology for conducting a meta-ethnography, which involves the following steps: 1. Getting started; 2. Deciding what is relevant; 3. Reading the studies; 4. Determining how the studies are related; 5. Translating studies into each other; 6. Synthesising translations; and 7. Expressing the synthesis [17]. Meta-ethnographies are widely used in healthcare research to form higher-level interpretations, rather than simply aggregating data, so this method was well-suited to the study aim. Reporting was structured using the eMERGe reporting guidance for meta-ethnographies [19].

Search strategy and process

Two authors (VP and BN) collaborated with information experts at Karolinska Institute University Library to develop comprehensive search strategies. Searches of 4 electronic databases (Medline; PsycINFO; Global Health; and Web of Science) were conducted from 10/06/2019 until 11/07/2019. The search strategy for Medline is presented in Supplementary Figure 1, other strategies are available upon request. Manual searches of citations on Google Scholar and reference lists were conducted to identify additional studies.

Qualitative studies exploring perceived barriers to care for migrant children and young people (aged up to 25 years) in HICs with mental or neurodevelopmental health problems were included. Studies exploring mental health problems; mental disorders or illness; and neurodevelopmental disorders or associated symptoms, were included. Studies exploring barriers to general care-seeking, or conducted in low or middle-income countries, were beyond the synthesis' scope and were not included. All years, study designs, and publication types were considered for inclusion. The search was limited to studies published in English.

Selecting primary studies

Study selection was an iterative process and search strategies were modified following new findings in the literature. Search results were imported into Mendeley reference management software for screening. Titles and abstracts were screened independently by VP and BN, who decided together on studies eligible for full-text screening. Full-text screens were carried out in the same manner. Disagreements were resolved by consultation with the research group.

Data extraction and determining how studies are related

VP and BN read studies independently, paying particular attention to the contexts in which studies were conducted. Next, first- and second-order constructs were extracted. Schutz [20] defined first-order constructs as participants' own interpretations, usually in the form of direct quotes, and second-order constructs as original authors' interpretations of participants' words, typically in the form of themes. VP and BN independently extracted first- and second-order constructs from the entirety of each study, creating a table of constructs that they discussed. Descriptive data was recorded in a data extraction form, first piloted on three studies by VP and EG.

Second-order constructs were compared across studies, identifying associations and differences to determine how studies were related. The table of extracted constructs was used to record authors' interpretations, which were discussed within the research group to challenge preconceptions and explore alternative interpretations.

Translation and synthesis

For peer review only

The following steps occurred in an iterative, non-linear process, but are described stepwise for clarity. Second-order constructs were translated into one another by VP, through comparison across all studies and grouping of constructs to develop new interpretations in the form of themes, representing perceived barriers to care. These themes are third-order constructs; researchers' interpretations of original authors' interpretations [21]. Discussion within the research group decided the final third-order constructs included in the synthesis.

The synthesis product, a line-of-argument synthesis [17], was developed by incorporating third-order constructs into Levesque's conceptual framework [18]. Thus, a version of the framework specific to our target population was developed. Finally, a narrative text synthesis was constructed by VP, ACH and KA, which expresses the third-order constructs in relation to the framework. Appropriate first-order constructs (quotes) were included to provide contextual richness.

Role of the funder

The funders of the study had no role in study design, data collection, data analysis, data interpretation, or writing of the report. The corresponding author had full access to all the data in the study and had final responsibility for the decision to submit for publication.

Patient and public involvement

No patient involved.

RESULTS

Included studies

The process of study selection is shown in Figure 2, which resulted in 30 studies being selected for inclusion [22,23,32–41,24,42–51,25–31].

Quality assessment

A quality assessment of included studies was carried out using an adapted version of the CASP (Critical Appraisal Skills Programme) quality assessment tool, presented in Table 1 [52]. Studies were not excluded based on these criteria, rather they were used to identify gaps in the quality of reporting [52]. Included studies were generally of good quality (Table 1), with four studies categorised as low-quality [27,37,40,44]. However, reporting of certain aspects was generally poor, such as researchers' role and justification of a qualitative approach.

Table 1. Quality criteria and results. Adapted version of the CASP quality assessment tool, taken from Atkins *et al*., 2008 [52].

Question	Yes	No	Unclear
Is this study qualitative research?	28	0	2
Are the research questions clearly stated?	24	6	0
Is the qualitative approach clearly justified?	20	7	3
Is the approach appropriate for the research question?	28	0	2
Is the study context clearly described?	27	2	1
Is the role of the researcher clearly described?	13	15	2
Is the sampling method clearly described?	27	1	2

For peer review only

Is the sampling strategy appropriate for the research question?	29	0	1
Is the method of data collection clearly described?	28	1	1
Is the data collection method appropriate to the research question?	26	1	3
Is the method of analysis clearly described?	25	4	1
Is the analysis appropriate for the research question?	27	2	1
Are the claims made supported by sufficient evidence?	27	1	2

Study characteristics

Study characteristics are presented in Table 2. Most studies were conducted in the USA, n=15 [22,30,47–51,31,33,37–39,41–43] or Australia, n=6 [26,27,29,34–36]. Individual interviews were the most common data collection method, n=17 [24,25,42,43,45,47,48,50,51,26,30,31,35,38–41], but focus group discussions, n=4 [22,27,28,32] or a combination of the two approaches, n=7 [23,29,33,34,36,46,49] were also employed. Fifteen included studies used thematic analysis [23,25,36,42,46,49,50,26,27,29–31,33–35], followed by content analysis, n=7 [28,32,40,41,45,47,48] and Grounded Theory, n=5 [22,24,37,39,51]. Of note, two articles were produced from the same data set but with different research questions [34,36], thus were considered separate studies. Five included studies are theses [45–48,51].

Study participants came from a variety of backgrounds (Table 2) and the majority of studies, n=22 [22,23,39–45,47–49,25,50,51,30,32–34,36–38] focused on specific migrant group(s). Study participants were parents/caregivers, n=10 [24,32,37,38,40–42,47,49,50], young people, n=6 [22,25,27,30,33,35], service providers, n=6 [29,31,39,43,48,51], or a combination of these, n=8 [23,26,28,34,36,44–46].

Table 2. Characteristics of included studies. ADHD – attention deficit hyperactivity disorder; ASD – autism spectrum disorders; AOD – alcohol and other drugs; CAMHS – Child and Adolescent Mental Health Services; FGD – focus group discussions; SBMHS – school-based mental health services; Latinx – person(s) of Latin American origin or descent. Note that the language used to describe participants was taken directly from included studies.

Study	Country	Type (no.) participants	Participant background (no.)	Data collection method; dates	Method of analysis	Study focus
Arora et al., 2019 [22]	USA	Adolescents aged 14-20 years (33)	First- and second-generation Asian-American	FGD; dates not reported	Grounded theory approach	To obtain information on Asian-American immigrant youth's perceptions of SBMH services and recommendations to address the mental health needs of this group

For peer review

Bradby et al., 2007 [23]	Scotland, UK	Parents (35), service users aged 6-14 years (7) and/or their parents (8), healthcare professionals (7), and carers (8) of children aged 5-13 years who had not been referred to CAMHS despite having suitable problems	South Asia	FGD and semi-structured interviews; September 2003 - March 2005	Thematic analysis	To explore attitudes to and experiences of CAMHS among families of South Asian origin who were underrepresented as service-users
Guzder et al., 2013 [24]	Canada	Parents (20) of children aged 7-12 years who attended a psychiatric day hospital for significant behaviour impairment	Immigrant (10) and native-born Canadian (10)	In-depth, semi-structured interviews; dates not reported	Grounded theory approach	To identify similarities and differences in perceptions of mental health problems and help seeking experiences between native-born and immigrant parents
Islam et al., 2017 [25]	Canada	Youth aged 15-23 years (10)	First- and second-generation South Asian	In-depth, semi-structured interviews; March-July 2015	Thematic analysis	To understand the mental health challenges and service access barriers experienced by South Asian youth populations
Posselt et al., 2017 [26]	Australia	Youth aged 12-25 years (15) and service providers (15): social workers, psychologists, and mental health nurses/nurse practitioners	Refugee youth from Afghan (9), African (4) and Bhutanese backgrounds (2); service providers background not stated	Semi-structured interviews; 2013-14	Thematic analysis	To determine the barriers and facilitators to effective, culturally responsive service provision for young people of refugee background with comorbid mental health and AOD problems

de Anstiss et al., 2010 [27]	Australia	Adolescents aged 13-17 years (85)	Refugee (various backgrounds)	FGD; dates not reported	Thematic analysis	To fill gaps in knowledge of rates and patterns of service utilisation across service sectors, use of informal supports, and actual and perceived barriers to mental health services amongst refugee adolescents
Goncalves et al., 2011 [28]	Portugal	Adolescents aged 12-17 (16), mothers (6), teachers and health professionals (17)	First- and second-generation immigrant adolescents and first-generation immigrant mothers (various backgrounds), teachers and health professionals background not stated	FGD; dates not reported	Content analysis	To shed light on the family-school-primary care triangle and the access to mental health care for migrant and ethnic minority families
Colucci et al., 2015 [29]	Australia	Service providers (115) from agencies that support refugee youth: mental health services, community support organisations, health services, schools, the state government health department, and a refugee resettlement agency	Not stated	FGD and semi-structured interviews; April 2010 - November 2011	Thematic analysis	To explore the perspectives of experienced service providers on barriers and facilitators to engaging refugee-background young people with mental health services

Wang et al., 2018 [42]	USA	Parents (19) of children aged 12-18 years	First-generation Asian	In-depth, semi-structured interviews; dates not reported	Thematic analysis	To investigate Asian immigrant parents' perception of barriers for seeking SBMHS for their adolescents
Forrest-Bank et al., 2019 [31]	USA	Service providers (14) from organisations serving refugee youth: community non-profit organisations, public schools, healthcare agencies, local government, an advocacy group, and a church.	Caucasian (12), not stated (2)	Semi-structured interviews; dates not reported	Inductive thematic analysis	To gain the perspectives of service providers about the strengths and barriers to mental health services addressing the needs of resettled refugee youth
Flink et al., 2012 [32]	The Netherlands	Mothers (41) of daughters aged 10-20 years	First-generation Moroccan (13) and Turkish (17), and Dutch (11)	FGD; dates not reported	Content analysis	To examine how mothers with different ethnic backgrounds perceive the issue of help-seeking for internalising problems experienced by adolescent girls
Ellis et al., 2010 [33]	USA	Adolescents aged 11-20 years (30)	First-generation Somali or Somali-Bantu	FGD and in-depth interviews; January 2004 - June 2006	Thematic analysis	To examine the utility of the Gateway Provider Model in understanding service utilization and pathways to help for Somali refugee adolescents
McCann et al., 2018 [34]	Australia	Youth aged 16-25 years (28), parents and	Sub-Saharan African youth, first-generation sub-Saharan	FGD and semi-structured interviews;	Inductive thematic analysis	To explore the stigma experience with mental illness and/or substance

		community leaders (41)	African parents, and community leaders	dates not reported		misuse amongst sub-Saharan African immigrants, and to examine the implications of this for help seeking for young people and parents from these communities
Valibhoy et al., 2017 [35]	Australia	Youth aged 18-25 years who had received services from a mental health professional in Australia (16)	Refugee (various backgrounds)	In-depth interviews; March 2012 - January 2013	Thematic analysis	To document the perspectives of youth from refugee backgrounds on their experience of accessing mental health services
McCann et al., 2016 [36]	Australia	Youth aged 16-25 years (28), parents and community leaders (41)	Sub-Saharan African youth, first-generation sub-Saharan African parents, and community leaders	FGD and semi-structured interviews; dates not reported	Inductive thematic analysis	To identify the help-seeking barriers and facilitators for anxiety, depression and alcohol and drug use problems in young people from recently established sub-Saharan African migrant communities
Gerdes et al., 2013 [37]	USA	Parents (73) of children aged 5-12 years	Latino	Problem recognition questionnaire for ADHD; dates not reported	Grounded theory	To better understand the help seeking process that occurs within Latino families when a child is exhibiting behaviours consistent with ADHD
Chapman et al., 2014 [38]	USA	Parents (16) of newly immigrated youth aged 12-18 years	First-generation Latino	Semi-structured interviews; August 2004 - March 2006	Narrative analysis	To examine parental perceptions of mental health and to determine patterns of help

						seeking and service use
Kang-Yi et al., 2018 [39]	USA	Church leaders (9) and early childcare workers (4)	First-generation Korean-American	Semi-structured interviews; September 2013 - August 2014	Grounded theory approach	To understand Korean immigrant families' and professionals' beliefs and attitudes towards autism and other developmental disorders
Shor, 2007 [40]	Israel and Russia	Parents in Israel (100) and Russia (100) of children under 18 years of age	Jewish immigrant parents from the Former Soviet Union (living in Israel) and Jewish parents living in Russia	Interviews; dates not reported	Content analysis	To differentiate between culturally-based help-seeking patterns of immigrant parents and those resulting from their new social context
AlAzzam & Daack-Hirsch, 2015 [41]	USA	Mothers (16) of children aged 5-12 years	First-generation Arab Muslim	Semi-structured interviews; dates not reported	Content analysis	To elicit Arab immigrant Muslim mothers' perceptions of and responses to behavioural problems in children (especially those associated with ADHD)
Wang et al., 2019 [30]	USA	Adolescents (55) aged 11-19 years	First- and second-generation Latinx- (25) and Asian-American (27), and bi-racial (Asian and Latinx, n=3)	Semi-structured interviews; 2014-15 academic year	Descriptive thematic analysis	To explore Asian– and Latinx–American adolescents' perceptions of seeking help for mental health concerns at middle or high schools
Ling et al., 2014 [43]	USA	Service providers (16) who work with Asian-American youth: counsellors, social workers,	White (3), Korean (4) and Chinese (9) ethnicity	In-depth, semi-structured interviews; March - June 2012	Consensual Qualitative Research method	To explore the perceived mental health needs of urban Asian-American adolescents and barriers to meeting their needs from the perspective of

		psychologists, organisation leaders and program coordinators, and an educator.				social service providers
Messent & Murrel, 2003 [44]	UK	Parents (7) who were attending or had attended CAMHS with their children, and social workers (4)	Bangladesh	Group meetings with semi-structured interview guide; dates not reported	None	To examine the accessibility of a child and adolescent mental health service to ethnic minority populations
Gill, 2010 [45]	Canada	Parents (4) of children aged 9-20 years, young adults aged 19-22 years (4), therapists and medical professionals (2), and a community leader (1)	First-generation Punjabi Sikh parents, second-generation Punjabi Sikh young adults, South-Asian mental health and medical professionals, and a Punjabi Sikh community leader	Semi-structured interviews; dates not reported	Content analysis	To describe the beliefs held by first-generation Canadian Punjabi Sikh parents about adolescent suicide and suicide-related behaviours
Choummanivong, 2013 [46]	New Zealand	Adolescents aged 13-18 years (53) and mental health service providers (20): psychologists, social workers, general practitioners, and school guidance counsellors	Refugee adolescents and health service providers from various backgrounds	FGD and semi-structured interviews; dates not reported	Thematic analysis	To provide more information about stressors impacting on refugee youth, their coping strategies, and their experience of mental health services in New Zealand
Nguyen, 2014 [47]	USA	Mothers (4) of children aged 3-12 years who had or were currently using mental	First-generation Vietnamese American	Semi-structured interviews; dates not reported	Content analysis (consistent with the multiple case study design)	To describe how Vietnamese American families that have used mental health services perceive and incorporate

		health services				children's mental health into their lives
Jacobs, 2014 [48]	USA	Mental health and human service providers who had experience working with Somali youth (8)	Somali (5) and Caucasian (3)	Semi-structured interviews; dates not reported	Content analysis	To explore how human service personnel view the barriers to services faced by Somali youth and how those barriers can be overcome
Zuckerman, 2014 [49]	USA	Parents (30) of typically developing children aged 2-10 years	First- and second-generation Latino	FGD and semi-structured interviews; dates not reported	Thematic analysis	To assess the understanding and conceptualisation of ASD in the Latino community in order to understand potential community barriers to early diagnosis
Araujo et al., 2017 [50]	USA	Caregivers (13) of children aged 7-10 years with ADHD symptoms that were receiving the Collaborative Life Skills program in school	Latino	Semi-structured interviews; dates not reported	Thematic analysis	To explore emotional, social, and cultural experiences of Latino youth with ADHD symptoms and their families
Smith, 2017 [51]	USA	Community members that provide services within communities with elevated rates of at-risk Latinx youth (11)	United States (9), Mexico (1) and Germany (1)	Qualitative interviews; December 2014 - March 2016	Grounded theory	To explore stressors and barriers to care in the Latinx youth community through a community-based participatory research framework

Study synthesis

Twenty third-order constructs, in the form of new themes representing barriers to care, were developed from first- and second-order constructs extracted from included studies. A worked example of the synthesis process is presented in Supplementary Table 1. The new themes were incorporated into

Levesque's conceptual framework of access to healthcare [18], through categorising them into the dimensions of abilities and accessibility that can influence the care-seeking process on the supply and demand side of care-seeking, as presented in Supplementary Table 2. The adapted framework, specific to barriers to care for our target population, is presented in Figure 3. The new themes are presented below within their respective dimensions of abilities and accessibility of the care-seeking process, on the supply and demand side of the care-seeking process.

Of note, the term "participants" indicates that a concept was discussed across different groups of participants in included studies - young people/children, caregivers, community members and service providers (Table 2). Concepts that were mentioned specifically by, or about, a particular group are specified as such.

Supply

Acceptability

Privacy and confidentiality concerns

Privacy and confidentiality concerns were identified as a significant barrier to seeking care for children's neurodevelopmental and mental health problems. Participants explained that in migrant communities, problems were often kept "in-house" to protect the family from the shame and heavy stigma attached to these conditions. One woman stated that in her community, "people will do nothing, only gossip" in the face of such problems. The consequences of disclosure were perceived as so damaging that they outweighed the benefits of care-seeking.

Similarly, young people expressed privacy concerns about school-based mental health services; they feared that information would be shared with teachers, peers, or parents, affecting their academic grades and social life. Parents worried that care-seeking would appear on the child's transcript and negatively impact their future. Some students, however, felt that school-based mental health services provided more privacy as they were detached from their community.

The fear of providers - particularly interpreters - breaching confidentiality was repeatedly identified as a barrier to care-seeking, as one young person explained:

"If I knew that person, and they knew my family, I wouldn't go near that place cos they might tell our parents."

Fear and mistrust of services

Closely linked to privacy concerns was a fear and mistrust of services. Parents explained that health and school systems were unfamiliar and therefore mistrusted, reducing the likelihood they would seek help there. Young people echoed this notion for school-based mental health services but emphasised a general mistrust of adults and mental health professionals as unable or unwilling to help.

Fear of authority and providing personal information were identified as significant barriers, particularly for refugee families. Service providers explained that these families were often fearful of the care-seeking process:

"For people who have been through certain traumas and come from countries with very difficult political situations, providing that amount of information on paper, in black and white on the referral form can be really confronting and a lot of people might be reluctant to do that with that information and not know where it's going or what it's going to be used for."

In addition, participants described a fear of retribution, such as deportation or involvement of social services. Another service provider explained:

For peer review only

"Lack of trust also is a big problem…they think if I tell them [service providers] this will they tell the police? Will they tell the child protection agencies?"

Negative perceptions and prior experiences of services

Perceptions and prior experiences of services were considered critical when deciding to seek care for children and young people. Negative past experiences of, and beliefs about, care-seeking spread through migrant communities, reducing the acceptability of seeking care. Furthermore, participants reported that services were perceived as overly reliant on medication, culturally unacceptable, and discriminatory. One young person stated:

"If you are born in Australia you get more respect- they [health services] care more about you because you are part of them, one of them. Rather than coming from overseas you get treated... [trails off]."

Young people described school-based mental health service providers as unable to relate to their concerns and not "real" mental health professionals, which formed a barrier to care-seeking. Adult members of migrant communities explained that mental health services were perceived as illegitimate and ineffective, giving "common sense" advice that family or friends could provide, or that service providers did not take their concerns seriously. Seeking care was therefore considered a "waste of time".

Availability and Accommodation

Location and appearance of services

Service providers in particular identified the location and appearance of services as a barrier to care-seeking for migrant young people. They described existing services as lacking the privacy needed due to the heavy stigmatisation of mental illness. One professional recalled:

"When I was in case work a client had to go to [a service] and when she walked out of the building, other people were actually waiting at a bus stop which was right in front and they said to her: "Why do you go in that place? That's for crazy people.""

Moreover, some providers described their facilities as too clinical or sterile, and inappropriate for migrant young people. The low appeal and stigmatisation of services was perceived as an obstacle to care-seeking.

Logistical and structural barriers

Participants identified logistical and structural barriers that migrant families faced to seeking care for children's neurodevelopmental disorders and mental health problems. Complex healthcare systems meant that families often found the many agencies and professionals involved confusing. Furthermore, complex referral processes, underfunded services, and long waiting times led to youth disengaging with or avoiding care-seeking. Appointment-based systems, restrictions on the number and length of appointments, and inflexible opening hours were also reported as barriers.

Some service providers also expressed frustration over strict eligibility criteria that excluded migrant young people who may benefit from services, as one provider recalled:

"I have found people over 25 who would fit into our youth program, and fit in very well, but because of the age limit, I can't offer them that, even though I think it would probably benefit them hugely."

Lack of information on mental health

Participants identified lack of information on mental health as a barrier to seeking care for children and young people's neurodevelopmental disorders and mental health problems. They reported that little or no information was available within migrant communities on the symptoms of mental illness

For peer review only

and importance of good mental health. If information was available, it was often not delivered in a way that was considered meaningful by members of these communities. Moreover, one youth participant described how the school curriculum focused solely on physical health:

"They only teach it or recognize physical unhealthy things in your body. Like if you are feeling sick or you feel nauseous or tummy is not feeling well…but there is never anything about if you feel like crying, if you feel very sad, if you feel very angry all the time."

This lack of information was believed to contribute to a lack of openness to seeking care.

Affordability

Associated costs deter help-seeking

The financial costs associated with accessing services were identified as a barrier to seeking care. Participants highlighted that migrant families often lacked the financial resources to seek care, identifying lack of time, insurance coverage, and money as barriers. Furthermore, some participants believed that mental health problems take "years to treat", incurring costs that families could not afford. Service providers highlighted loss of income as a key deterrent:

"Refugee families are working really hard you know, and income is absolutely essential for them… and if we're going to be calling them away from their work schedule, that's a loss of income for them in order to attend these appointments."

Participants in certain settings identified the cost of treatment as a barrier, explaining that it was not always affordable for migrant families. Others highlighted that perceived cost deterred care-seeking. They explained that in some countries, specialist care was a luxury few could afford. Even if this was not the case in their new homeland, the misconception stopped families from seeking care.

The stressors associated with poverty were identified as a further barrier to care-seeking by providers; they reported that when money was short, young people could feel pressured to drop out of school to support the family. This was perceived to isolate young people from community resources, reducing the likelihood that they would seek help if needed.

Appropriateness

Lack of information and services in other languages

Language and communication issues were identified as a barrier to migrant families seeking and engaging with services. Participants from migrant communities reported that information was often not available in their mother tongue or was of poor quality, while service providers stated that there was no information suitable for those with a low literacy level, stopping them from accessing information and services.

A shortage of bilingual service providers was perceived as a barrier in various studies, as this left providers and families reliant on interpreters. Providers emphasised that the availability of interpreters varied, with some services only available by phone, or that the quality of services was low. This was perceived to damage family-provider relationships and discourage further care-seeking within communities. Indeed, not all providers were willing to use interpreters. One provider expressed their frustration:

"Most of our interpreters they… don't have enough experience. So, when people become proficient they leave for another job and leave this to a beginner. And the beginners they cannot make the things effective. So sometimes they mislead the people more often than are helpful."

Interpreting services for newer migrant communities were reported to be of poorer quality, forming a further barrier to care for newly arrived families.

For peer review only

Service providers lack cultural responsiveness

Lack of cross-cultural responsiveness among service providers was perceived to limit the value of help-seeking for children and young people with neurodevelopmental disorders or mental health problems. In contrast to concerns over confidentiality, participants from migrant communities expressed a preference for same-culture service providers, who would understand their values and experiences. These participants emphasised that service providers were lacking in knowledge of the communities they serve, while providers themselves expressed frustration at their insecurities and lack of insight when working with migrant communities: "I'm walking into this almost blind." Moreover, providers lacking cross-cultural awareness were viewed as judgemental of migrant families. One mother shared an anecdote from her community:

"[T]he girl went to the counsellor and the counsellor started asking how you were treated when you were young, some routine questions. Then halfway through, the counsellor was shocked by some of the difficult ways or how the parenting practices [are] normal…He basically thought this girl was abused, emotionally…so the girl, instead of getting help with her mental illness, she started to defend her parents."

Demand

Ability to perceive a need for care

Limited knowledge of hosting country's model of mental and neurodevelopmental health

Lack of exposure to a "Western", or biopsychosocial, model of mental and neurodevelopmental health was identified as a barrier to seeking care within migrant communities. This was described to contribute to low mental health literacy and problem recognition. Participants in various studies, expressed a different understanding of the concept of mental health. In one study, for example, the group facilitator explained the meaning of mental health within the hosting country to a group of young people:

"Okay, you know back home? Mental health – people are crazy who dance (in the street) and shit? Aha! Those are the people we are talking about. Those ones who run around. Over there, we don't call them mental, just people who are crazy…But here it's different. Somebody might not dance in the street. They might be dressed in a suit and still have a mental problem … So now you get the picture?"

A general lack of awareness of mental health within migrant communities was also reported. Participants attributed this to various causes, including that mental health was "taboo", and that these conditions were not visible and thus difficult to conceptualise. Some parents of children with mental illness described feeling bewildered at the onset of their children's suffering, which they had not initially recognised as illness. Other parents described difficulty distinguishing between normal and abnormal behaviour.

Participants both reported and held misconceptions surrounding mental health and neurodevelopmental disorders. These included that they do not affect children; are lifelong; and are only present within certain communities. One young person stated:

"I've never met an African suffering from anxiety. Depression is not very common in my community."

Alternative explanatory models of illness

Alternative explanatory models of mental and neurodevelopmental disorders, which differed from the conceptualisation of these conditions in hosting countries, were reported to impede professional care-seeking within migrant communities. Mental illness was described by some migrant participants as contagious; while others believed that mental illness was due to a curse or possession by an evil spirit,

For peer review only

thus care would be sought from witchdoctors or spiritual leaders. Another prevalent idea was that a child's problems reflected the family's faults, so care-seeking was avoided to protect the family's standing within the community. One church leader explained:

"Koreans – even if they're living in the US – tend to think that a child's [neurodevelopmental] disorder is the result of someone wrongdoings, or something bad that runs in the family. As a matter of fact, until recently, and even now, many Koreans avoid marrying someone from a family with a disabled person."

In addition, participants reported that these conditions were attributed to poor family relations, children who were "spoiled" or lacked discipline. Some members of the migrant community stated that mental illness was due to over-protection of young people in "Western" societies:

"Back home, we never saw this behaviour with mental health among younger people. It's happening [here] because the government endorses those behaviours, because kids are protected. It's something [we've] never seen back home."

Ability to seek

Stigma

The stigma attached to neurodevelopmental disorders and mental health problems in migrant communities was repeatedly highlighted as a barrier to seeking care, across groups of participants. These conditions were associated with the "shame mark" and embarrassment, thus seeking help outside the family was avoided to prevent rumours and community rejection. Some migrant community members stated that seeking help from trusted, informal sources would minimise the risk of gossip. However, stigma was cited as a barrier for community members (such as childcare workers) to suggest that a family seek help for their child, because this could be interpreted as an insult.

Participants reported that mental illness and neurodevelopmental disorders were so stigmatised that diagnosis would damage a child's marriage prospects and the family's reputation. Even when participants recognised the benefits of seeking care, the costs were deemed too high:

"The parents have to find a daughter-in-law for themselves. That's the real point. Like no one, no one, will accept the proposal of crazy guy."

This stigma extended to care-seeking, which was described as a sign of weakness and was associated with negative stereotypes including being "psycho". Moreover, some young people stated that their parents had not sought care for their own mental health problems, which reinforced internalised stigma towards help-seeking:

"Because I realized later she [mother] had postpartum depression and then suffered more depression after I was born and then because it all gets ignored within my family, it all just carries on. And then 10 years later she was just battling general depression. Then my sister was born, then it was postpartum depression. It was just a cycle."

Difficulty Accepting Illness

Participants reported that the stigma attached to neurodevelopmental and mental health problems made it difficult to accept that a child was ill, leading to denial that formed a barrier to care-seeking. Some participants reported that denial allowed parents to continue to believe that their child was "perfect":

"[Vietnamese parents] Don't want to look and admit that their children are sick. Their children need help and will not admit it. They say that their children are beautiful, just like that."

Young people's denial of a problem was also highlighted as a barrier to care-seeking; adults and young people alike reported that adolescents hid their problems due to embarrassment or pride. This

For peer review only

hindered independent care-seeking and made it difficult for parents to recognise a problem and initiate the care-seeking process.

Preference for self-support

Both adults and young people identified a preference for self-support as a barrier to seeking professional care. One's problems were viewed as a burden that should not be placed on others; a view that was particularly prevalent amongst young people, but also stopped parents from seeking support from other family members or health professionals.

According to some parents, young people from migrant backgrounds preferred self-support as they want to feel more "adult" and independent. Young people themselves reasoned that everyone experienced these types of problems, thus they should be able to cope alone. One participant stated:

"I didn't wanna tell anyone 'cause I didn't think I had a problem, and I could fix it on my own. So, I feel like a lot of kids feel like that…That they can just fix it on their own."

In addition, participants reported that young people feared being coerced into seeking care if they discussed their problems with their parents. This fear of losing control formed a barrier to problem-sharing and thus care-seeking.

Cultural values deter help-seeking

Certain cultural values within migrant communities were perceived as a barrier to help-seeking for neurodevelopmental disorders and mental health problems. Participants form migrant communities expressed a preference for informal support, such as friends or churches, explaining that those who knew the child were better equipped to help. Mental health services were often described as a last resort.

Moreover, participants identified prioritising academic success over mental health as a barrier to care-seeking. Students from Asian communities reported that a fear of not living up to the "model minority" stereotype could deter their peers from seeking help for mental health problems. Other perceived barriers included a culture of not complaining, and low priority placed on mental health and individual needs. Latinx (person(s) of Latin American origin or descent) participants emphasised how central *familismo* (the importance of the family) is in their culture. Young people were expected to prioritise the family's needs over their own, including their mental health. One young person stated:

"You don't talk about emotions, you don't deal with mental health, it doesn't exist. You just need to get a job, go to school, do what you need to do, pay your bills, buy your house. All of that. You just ignore mental health."

Ability to reach

Immigration and legal status

Concerns over immigration and legal status were identified as important barriers to care within migrant communities, including amongst those residing legally. Participants explained that (particularly undocumented) migrants feared that seeking mental health care would lead to deportation, while some incorrectly assumed that refugees were not entitled to these services. This led some parents to minimise contact with government organisations, impeding care-seeking for their children. One participant explained:

"Going to family doctor, as an African migrant, we are scared to put somebody in the system, like if it is at early stage of mental illness, because everything here is documented. If you are on the system, it will be very hard to get rid of it."

Limited knowledge of services

For peer review only

Limited knowledge of the mental health care system (and, occasionally, no equivalent system in country of origin) made it difficult for migrant families to understand how to seek care for their children. One young person noted, “Even if they were kind of aware of it [services], they wouldn’t know where to start.”

Similarly, participants emphasised that knowledge of the types of healthcare providers was often low within migrant communities, thus families were unsure where to seek help. However, participants noted that second-generation migrants were often better-informed and more likely to seek care than newly arrived migrants.

Participants described common misconceptions surrounding who could seek mental health services. Young people, for example, incorrectly stated that school-based mental health services were only for severe mental illness or academic concerns.

Lack of familial support

Lack of familial support and communication were identified as important barriers to care-seeking, preventing children and young people from reaching care. Adolescents described how discussing their mental health with their parents resulted in dismissive responses, which deterred further communication. Some explained that their parents did not understand, while others said that parents were more concerned about their academic grades.

Similarly, participants reported about communication barriers within migrant families. Disagreeing with parents was not always culturally acceptable, which prevented open discussions around mental health. In addition, some young people said that their parents handled their emotions in an unhealthy way and treated help-seeking with disdain. One participant described her father’s struggles to express his feelings:

“I feel like my dad, is just like, the way, traditional thinking of Pakistani men. Right? Unfortunately. Like it has nothing to do with oh, I will miss you or anything. [i.e. he does not express how much he loves his children.]”

Ability to engage

Language and communication barriers

Limited language proficiency was identified as a barrier to families engaging with services; participants highlighted that parents with poor language skills lacked the confidence to communicate with schools and service providers, preventing them from benefitting from available resources. Although young people were considered to have fewer difficulties, this was not always the case - some young people reported struggling to express concepts related to mental health in their new language. Some concepts did not directly translate into all languages, which formed a communication barrier between families and providers, but also between parents and children with different levels of language proficiency. One participant from a migrant community explained:

“We don’t really have words [in Somali], like a word for depression or anxiety, but we have words like someone being stressed out, worrying a lot…but those are just temporary states though, it’s not like over a long period of time. So it’s like I’m stressed out or she’s stressed out lately, but something continuing for a long period of time, we don’t have anything like that.”

DISCUSSION

Summary of findings

Our qualitative synthesis produced twenty overarching themes, representing perceived barriers to seeking care for children and young people’s neurodevelopmental and mental health problems within

For peer review only

migrant communities in HICs (Figure 3). These themes were used to modify Levesque's conceptual framework of patient-centred access to healthcare [18], creating a version of this framework specific to our target population, presented in Figure 3. We felt that the linearity and unidirectionality of the original framework (Figure 1) did not reflect the dynamicity of the care-seeking process for migrant children and young people, as expressed by study participants. Similarly, barriers to care were not assigned to one particular step in the care-seeking pathway (Figure 1). Our modified framework therefore depicts barriers to care surrounding a circular care-seeking pathway, encapsulating its complexity and non-linearity (Figure 3).

To our knowledge, this is the first synthesis of qualitative data on perceived barriers to care for children and young peoples' neurodevelopmental and mental health problems within migrant communities. The adapted framework provides a snapshot of the perceived barriers to seeking care from the perspective of those who experience them, while highlighting that these barriers exist and interact within the context of communities and systems. Given the heterogeneity of settings and migrant groups included in the synthesis, we envision that the framework can be used as a tool (alongside in-depth knowledge of local contexts) to inform policy and service provision on migrant children and young people's mental health. We invite researchers to comment on and expand the framework to improve its utility within this field.

Most included studies were conducted in the USA (n=15) or Australia (n=6); unsurprising given that both were in the top 20 destinations for all migrants in 2019 [53]. However, a lack of studies from other countries such as the UK (n=1) was unexpected and could indicate a lack of research in this area, despite high numbers of resident migrants [53]. Similarly, the number of included studies was lower than expected, which could indicate a need for more qualitative research into how people in migrant communities experience and perceive barriers to children and young people's mental health care. The most common participants were parents and caregivers (n=10), likely linked to their role as gatekeepers to seeking care. Moreover, eight studies included multiple different groups of participants (Table 2), which can capture different dimensions of the care-seeking process [54].

The fact that 22 studies focused on perceived barriers within specific migrant group(s) may reflect a move away from a "one size fits all" approach to migrant health. However, a key finding of the synthesis is that the barriers to care were repeatedly identified across study participants (parents, young people, service providers, and different migrant communities), on the supply and demand side of the care-seeking process (Figure 3). This suggests that different migrant communities face common difficulties in seeking mental health care for children and young people in HICs. A recent systematic review found that refugees, asylum seekers, and undocumented migrants in Europe faced common (and very similar) barriers to care, including lack of knowledge of healthcare systems and trust in health professionals [5], supporting our findings. There is currently no systematic review or meta-analysis on barriers to mental health care for migrant children and young people, however, which could further strengthen our findings.

Furthermore, many of the barriers to seeking care for migrant children and young people have also been identified in general populations [55]. A recent systematic review of qualitative and quantitative studies on barriers to mental health care amongst children and adolescents (which included two studies in this synthesis [27,28]) reported many similar barriers, including: lack of knowledge of mental health and mental health services, preference for self-reliance, and perceived effectiveness of professional help [56]. This suggests that these barriers are associated with children and young people's care-seeking, or care-seeking for stigmatised conditions, such as mental health [57]. Indeed, several barriers identified in this synthesis (such as lack of familial support or difficulty accepting illness) have been linked to the stigmatisation of mental health amongst children and young people in HICs [56].

Our findings suggest that migrant families often perceive multiple barriers to care, several of which are likely specific to migrant communities or ethnic minorities, including: language and

communication barriers, immigration and legal status, and lack of cultural responsiveness among providers (Figure 3). These additional barriers to seeking mental or neurodevelopmental health care for migrant children and young people may contribute to low care-seeking amongst this group [5,16]. Interventions targeting multiple barriers may be needed to engage migrant families in the care-seeking process.

Caregivers, young people, and service providers expressed frustration at the lack of bicultural providers and lack of cultural responsiveness among service providers. However, interpreters from one's own community were considered likely to spread gossip, highlighting how barriers to care can interact and difficulties to overcoming them. Cultural sensitivity training for providers is one option to improve relationships with migrant communities and increase care-seeking, but the evidence for this is limited [60] and alone it is unlikely to address the diversity of cultures and practices within different migrant communities.

Stigmatisation of mental and neurodevelopmental health forms a common thread through the results of this synthesis, connecting barriers such as fear and mistrust of services, and confidentiality concerns. The stigma associated with mental health has been associated with reduced care-seeking within migrant populations [14,59]. Our findings support the notion that stigma exists at many levels, fostering misunderstanding and mistrust of services and professionals [60]. Tackling stigma is complex, likely requiring long-term interventions to improve health literacy, alter attitudes and behaviour. A recent systematic review of reviews on interventions to reduce stigma related to mental health and suicide in general populations found that some interventions were successful at improving knowledge and attitudes in the short-term, but there was a lack of evidence on interventions for stigma surrounding less severe mental health problems [62]. Although studies have attempted to tackle stigma to increase mental health care-seeking within migrant communities [62,63], more research on how to effectively reduce this stigma amongst migrant children and young people is needed.

Limitations and methodological considerations

This study has several limitations. The relatively large number of included studies (n=30) for a qualitative synthesis, with diverse settings and participants, may have reduced the analytical depth of the meta-ethnography. However, given that similar barriers were identified across studies, this effect is judged to be minimal. The quality assessment of included studies was not used to exclude studies, reducing the trustworthiness of findings. Our search strategy was limited to studies published in English, which may partly explain the high number of studies from English-speaking countries (n=27), a limitation of this study. Moreover, although a broad research aim and comprehensive search strategy were employed, it was not possible to search all relevant databases and journals, thus relevant studies may have been missed.

A meta-analysis of qualitative and quantitative data was not conducted, which could provide evidence on how barriers to care affect care-seeking and allow comparison between studies on perceived and actual barriers to care. However, increasing our understanding of how migrant families perceive and experience barriers to care through a qualitative synthesis is a critical first step to overcoming these challenges.

In qualitative research, the trustworthiness of a methodology is commonly assessed using four criteria: credibility, dependability, confirmability and transferability [64]. Credibility was achieved through the use of the eMERGe reporting guidelines [19]. Authors kept a diary throughout the process to record their reflections and held regular de-briefs to reflect on their interpretations of the data, further enhancing credibility. Dependability was attained through multiple authors independently carrying out steps of the methodology and meeting to confer. The varied backgrounds and expertise within the research group enabled authors to explore different aspects of the data, while still agreeing upon the line-of-argument synthesis, increasing the robustness of findings. Further, these varied backgrounds

For peer review only

allowed the research team to question one another's interpretations, increasing dependability. Confirmability was enhanced by researchers acknowledging their preconceptions of the barriers to care, while reflecting on their position and preconceptions throughout the study.

Implications for practice and future research

The similarities in perceived barriers to mental and neurodevelopmental health care between children and young people in general and migrant populations suggests that broader public health interventions, such as increased mental health education in schools or public awareness campaigns, could increase care-seeking across the groups. However, our results demonstrate that migrant communities perceive further, specific barriers to care, which must be carefully mapped to ensure that interventions are adapted where required.

Our results demonstrate a need to increase trust in mental health services within migrant communities in HICs. Improving communication and encouraging discussion of neurodevelopmental and mental health could reduce the fear and stigma associated with help-seeking. Increasing numbers of bicultural practitioners and improving cultural responsiveness, alongside these interventions, will be critical to increase trust and ensure that communities engage with interventions.

This meta-ethnography highlights several gaps in the field. While our findings suggest that perceived barriers are broadly similar between children and young people in general populations and different migrant communities, this should be confirmed through a meta-analysis of quantitative and qualitative data. This can identify any differences between these groups in perceived barriers and service access, to better inform policy and intervention design. In addition, more studies exploring how migrant communities perceive and experience barriers to care is essential. Finally, research on interventions that facilitate care-seeking for migrant children and young people's mental and neurodevelopmental health problems are much needed. This should ideally involve input from migrant community members, to ensure that interventions are well-suited to the communities they seek to reach.

Conclusion

This meta-ethnography synthesised the findings of 30 qualitative studies examining perceived barriers to seeking care for migrant children and young people with neurodevelopmental and mental health problems in HICs. Findings demonstrate that many of the perceived barriers to care were identified across different types of study participants and migrant groups, and are similar to those perceived by children and young people in general populations. However, migrant families often face further, specific barriers to care. These findings demonstrate the dynamic nature of care-seeking, which presents a challenge to designing effective policy and interventions to ensure that families reach services. Our adapted conceptual framework can help professionals and policy makers to visualise the complex nature of the barriers migrant families face to seeking care within the wider system context, and assist in improving practice and interventions to overcome these barriers. However, much further research is required to determine how migrant status affects the barriers to care faced by children and young people, and effective interventions to overcome them. As international migration continues to rise, and the COVID-19 pandemic is expected to trigger a further increase in the number of people fleeing poverty across borders, ensuring that migrant children and young people reach and benefit from mental health care is more vital than ever.

Author Contributions

CD and ACH conceived the project. VP and BN carried out the database searches, screened titles, abstracts, and full texts, and extracted data together with EG. VP conducted the synthesis and wrote the manuscript with contributors. ACH provided supervision to VP and critically revised the manuscript at all stages. All authors reviewed the study findings and read and approved the final version before submission.

For peer review only

Funding

Swedish research council (Vetenskapsrådet) grant nr: 2018-05763.

ACH was funded by Forte 2016-00870

Competing interests

None declared.

Ethics statement

All data included in this article is secondary data, already published with ethical approval hence no additional permission is needed.

Data sharing statement

Not applicable.

FIGURE LEGENDS

Figure 1. A conceptual framework of patient-centred access to healthcare. Adapted from Levesque *et al.*, 2013 [19].

Figure 2. Study selection. HIC – high-income country.

Figure 3. A conceptual framework of patient-centred access to mental health care for migrant children and young people. Adapted from Levesque *et al.*, 2013 [19].

Supplementary Figure 1. Search strategy for Medline.

REFERENCES

1 United Nations Department of Economic and Social Affairs (Population Division). International Migration 2019: Report (ST/ESA/SER.A/438). New York: 2019.

2 Bhopal RS. COVID-19: Immense necessity and challenges in meeting the needs of minorities, especially asylum seekers and undocumented migrants. *Public Health* 2020;**182**:161–2. doi:10.1016/j.puhe.2020.04.010

3 United Nations Department of Economic and Social Affairs Population Division. International Migrant Stock 2019. 2019. https://www.un.org/en/development/desa/population/migration/data/estimates2/countryprofiles.asp

4 UNHCR. Figures at a Glance. 2020.https://www.unhcr.org/figures-at-a-glance.html (accessed 24 Jun 2020).

5 Priebe S, Giacco D, El-Nagib R. Public health aspects of mental health among migrants and refugees : a review of the evidence on mental health in the WHO European Region. Copenhagen: 2016.

6 Fazel M, Reed R V., Panter-Brick C, *et al.* Mental health of displaced and refugee children resettled in high-income countries: Risk and protective factors. *Lancet* 2012;**379**:266–82. doi:10.1016/S0140-6736(11)60051-2

7 Kim Y, Evans BE, Hagquist C. Mental Health Problems Among Adolescents in Sweden from 1995 to 2011: The Role of Immigrant Status and the Proportions of Immigrant Adolescents in Their Surrounding Community. *J Immigr Minor Heal* 2020;**22**:232–9. doi:10.1007/s10903-019-00951-0

For peer review only

8 Vervliet M, Meyer Demott MA, Jakobsen M, *et al.* The mental health of unaccompanied refugee minors on arrival in the host country. *Scand J Psychol* 2014;**55**:33–7. doi:10.1111/sjop.12094

9 Lindström M, Rosvall M, Modén B. Country of birth, parental background and self-rated health among adolescents: A population-based study. *Scand J Public Health* 2014;**42**:743–50. doi:10.1177/1403494814545104

10 Bronstein I, Montgomery P. Psychological Distress in Refugee Children: A Systematic Review. *Clin Child Fam Psychol Rev* 2011;**14**:44–56. doi:10.1007/s10567-010-0081-0

11 Manhica H, Almquist Y, Rostila M, *et al.* The use of psychiatric services by young adults who came to Sweden as teenage refugees: A national cohort study. *Epidemiol Psychiatr Sci* 2017;**26**:526–34. doi:10.1017/S2045796016000445

12 Jablonska B, Kosidou K, Ponce de Leon A, *et al.* Neighborhood Socioeconomic Characteristics and Utilization of ADHD Medication in Schoolchildren: A Population Multilevel Study in Stockholm County. *J Atten Disord* 2020;**24**:265–76. doi:10.1177/1087054716643257

13 Brendler-Lindqvist M, Norredam M, Hjern A. Duration of residence and psychotropic drug use in recently settled refugees in Sweden - a register-based study. *Int J Equity Health* 2014;**13**.http://www.equityhealthj.com/content/pdf/s12939-014-0122-2.pdf

14 Satinsky E, Fuhr DC, Woodward A, *et al.* Mental health care utilisation and access among refugees and asylum seekers in Europe: A systematic review. *Health Policy (New York)* 2019;**123**:851–63. doi:10.1016/j.healthpol.2019.02.007

15 Colucci. The utilisation of mental health services by children and young people from a refugee background: A systematic literature review. Int. J. Cult. Ment. Health. 2014;**7**:86.

16 Markkula N, Cabieses B, Lehti V, *et al.* Use of health services among international migrant children - a systematic review. *Global Health* 2018;**14**:52. doi:10.1186/s12992-018-0370-9

17 Nobilt G, Hare R. *Meta-ethnography: synthesizing qualitative studies*. California: : SAGE Publications Inc 1988.

18 Levesque J-F, Harris M, Russell G. Patient-centred access to health care. *Int J Equity Health* 2013;**12**:1–9. doi:10.1002/cber.189502803178

19 France EF, Cunningham M, Ring N, *et al.* Improving reporting of meta-ethnography: The eMERGe reporting guidance. *BMC Med Res Methodol* 2019;**19**:1–13. doi:10.1186/s12874-018-0600-0

20 Schutz A. *Collected Papers I: The Problem of Social Reality*. 1962.

21 Britten N, Campbell R, Pope C, *et al.* Using meta ethnography to synthesise qualitative research: A worked example. *J Heal Serv Res Policy* 2002;**7**:209–15. doi:10.1258/135581902320432732

22 Arora PG, Algios A. School-Based Mental Health for Asian American Immigrant Youth: Perceptions and Recommendations. *Asian Am J Psychol* 2018;**10**:166–81. doi:10.1037/aap0000142

23 Bradby H, Varyani M, Oglethorpe R, *et al.* British Asian families and the use of child and adolescent mental health services: A qualitative study of a hard to reach group. *Soc Sci Med* 2007;**65**:2413–24. doi:10.1016/j.socscimed.2007.07.025

24 Guzder J, Yohannes S, Zelkowitz P. Helpseeking of immigrant and native born parents: A qualitative study from a montreal child day hospital. *J Can Acad Child Adolesc Psychiatry* 2013;**22**:275–81.http://ovidsp.ovid.com/ovidweb.cgi?T=JS&PAGE=reference&D=prem1&NEWS=N&AN=

For peer review only

24223046

25 Islam F, Multani A, Hynie M, *et al.* Mental health of South Asian youth in Peel Region, Toronto, Canada: A qualitative study of determinants, coping strategies and service access. *BMJ Open* 2017;**7**:e018265. doi:10.1136/bmjopen-2017-018265

26 Posselt M, McDonald K, Procter N, *et al.* Improving the provision of services to young people from refugee backgrounds with comorbid mental health and substance use problems: Addressing the barriers. *BMC Public Health* 2017;**17**:280. doi:10.1186/s12889-017-4186-y

27 de Anstiss H, Ziaian T. Mental health help-seeking and refugee adolescents: Qualitative findings from a mixed-methods investigation. *Aust Psychol* 2010;**45**:29–37. doi:10.1080/00050060903262387

28 Gonçalves M, Moleiro C. The family-school-primary care triangle and the access to mental health care among migrant and ethnic minorities. *J Immigr Minor Heal* 2012;**14**:682–90. doi:10.1007/s10903-011-9527-9

29 Colucci E, Minas H, Szwarc J, *et al.* In or out? Barriers and facilitators to refugee-background young people accessing mental health services. *Transcult Psychiatry* 2015;**52**:766–90. doi:10.1177/1363461515571624

30 Wang C, Barlis J, Do KA, *et al.* Barriers to Mental Health Help Seeking at School for Asian– and Latinx–American Adolescents. *School Ment Health* Published Online First: 2019. doi:10.1007/s12310-019-09344-y

31 Forrest-Bank SS, Held ML, Jones A. Provider Perspectives of Services Addressing the Mental Health Needs of Resettled Refugee Youth. *Child Adolesc Soc Work J* Published Online First: 2019. doi:10.1007/s10560-019-00602-1

32 Flink IJE, Beirens TMJ, Butte D, *et al.* The role of maternal perceptions and ethnic background in the mental health help-seeking pathway of adolescent girls. *J Immigr Minor Heal* 2013;**15**:292–9. doi:10.1007/s10903-012-9621-7

33 Ellis BH, Lincoln AK, Charney ME, *et al.* Mental Health Service Utilization of Somali Adolescents: ReligioN, Community, and School as Gateways to Healing. *Transcult Psychiatry* 2010;**47**:789–811. doi:10.1177/1363461510379933

34 McCann T V., Renzaho A, Mugavin J, *et al.* Stigma of mental illness and substance misuse in sub-Saharan African migrants: A qualitative study. *Int J Ment Health Nurs* 2018;**27**:956–65. doi:10.1111/inm.12401

35 Valibhoy MC, Kaplan I, Szwarc J. 'it comes down to just how human someone can be': A qualitative study with young people from refugee backgrounds about their experiences of Australian mental health services. *Transcult Psychiatry* 2017;**54**:23–45. doi:10.1177/1363461516662810

36 McCann T V, Mugavin J, Renzaho A, *et al.* Sub-Saharan African migrant youths' help-seeking barriers and facilitators for mental health and substance use problems: A qualitative study. *BMC Psychiatry* 2016;**16**:275. doi:10.1186/s12888-016-0984-5

37 Gerdes AC, Lawton KE, Haack LM, *et al.* Latino parental help seeking for childhood ADHD. *Adm Policy Ment Heal Ment Heal Serv Res* 2014;**41**:503–13. doi:10.1007/s10488-013-0487-3

38 Chapman M V., Stein GL. How do new immigrant Latino parents interpret problem behavior in adolescents? *Qual Soc Work* 2014;**13**:270–87. doi:10.1177/1473325012468478

39 Kang-Yi CD, Grinker RR, Beidas R, *et al.* Influence of Community-Level Cultural Beliefs about Autism on Families' and Professionals' Care for Children. *Transcult Psychiatry* 2018;**55**:623–47. doi:10.1177/1363461518779831

For peer review only

40 Shor R. Differentiating the culturally-based help-seeking patterns of immigrant parents from the former Soviet Union by comparison with parents in Russia. *Am J Orthopsychiatry* 2007;**77**:216–20. doi:10.1037/0002-9432.77.2.216

41 AlAzzam M, Daack-Hirsch S. Arab Immigrant Muslim Mothers' Perceptions of Children's Attention Deficit Hyperactivity Disorder. *Procedia - Soc Behav Sci* 2015;**185**:23–34. doi:10.1016/j.sbspro.2015.03.454

42 Wang C, Do KA, Frese K, *et al.* Asian Immigrant Parents' Perception of Barriers Preventing Adolescents from Seeking School-Based Mental Health Services. *School Ment Health* 2019;**11**:364–77. doi:10.1007/s12310-018-9285-0

43 Ling A, Okazaki S, Tu MC, *et al.* Challenges in Meeting the Mental Health Needs of Urban Asian American Adolescents: Service Providers' Perspectives. *Race Soc Probl* 2014;**6**:25–37. doi:10.1007/s12552-014-9117-2

44 Messent P, Murrell M. Research Leading to Action: A Study of Accessibility of a CAMH Service to Ethnic Minority Families. *Child Adolesc Ment Health* 2003;**8**:118–24. doi:10.1111/1475-3588.00057

45 Iqbal Kaur G. *Punjabi sikh parents' beliefs about suicide and suicide-related behaviours*. 2010.

46 Choummanivong C. *Refugee Youths: Adaptation and Mental Health Service Provision*. 2013.

47 Nguyen NH. *Vietnamese American Families' Perceptions of Children's Mental Health: How Culture Impacts Utilization of Mental Health Services*. 2014.

48 Jacobs M. *Better Mental Health Service Provision for Somali Youth: Overcoming the Barriers*. 2014.

49 Zuckerman KE, Sinche B, Cobian M, *et al.* Conceptualization of Autism in the Latino Community and its Relationship With Early Diagnosis. *J Dev Behav Pediatr* 2014;**35**:522–32. doi:10.1097/DBP.0000000000000091

50 Araujo EA, Pfiffner L, Haack LM. Emotional, Social and Cultural Experiences of Latino Children with ADHD Symptoms and their Families. *J Child Fam Stud* 2017;**26**:3512–24. doi:10.1007/s10826-017-0842-1

51 Smith LT. 'They might not show it and they might not say it, but in their mind they're really stressed' : a qualitative exploration of stressors and barriers to care for Latinx youth. Published Online First: 2017.https://repositories.lib.utexas.edu/bitstream/handle/2152/47450/SMITH-MASTERSREPORT-2017.pdf

52 Atkins S, Lewin S, Smith H, *et al.* Conducting a meta-ethnography of qualitative literature: Lessons learnt. *BMC Med Res Methodol* 2008;**8**:1–10. doi:10.1186/1471-2288-8-21

53 International Organisation for Migration. World Migration Report 2020. Geneva: 2020. doi:10.1002/wom3.2

54 Green J, Thorogood N. *Qualitative Methods for Health Research*. 4th ed. London: : SAGE Publications Ltd STM 2018.

55 Gulliver A, Griffiths KM, Christensen H. Perceived barriers and facilitators to mental health help-seeking in young people: a systematic review. *BMC Psychiatry* 2010;**10**:113. doi:10.1186/1471-244X-10-113

56 Radez J, Reardon T, Creswell C, *et al.* Why do children and adolescents (not) seek and access professional help for their mental health problems? A systematic review of quantitative and qualitative studies. *Eur Child Adolesc Psychiatry* Published Online First: 2020. doi:10.1007/s00787-019-01469-4

For peer review only

57 Corrigan PW, Watson AC. Understanding the impact of stigma on people with mental illness. *World Psychiatry* 2002;**1**:16–20.https://pubmed.ncbi.nlm.nih.gov/16946807

58 Jongen C, McCalman J, Bainbridge R. Health workforce cultural competency interventions: a systematic scoping review. *BMC Health Serv Res* 2018;**18**:232. doi:10.1186/s12913-018-3001-5

59 Byrow Y, Pajak R, Specker P, *et al.* Perceptions of mental health and perceived barriers to mental health help-seeking amongst refugees: A systematic review. *Clin Psychol Rev* 2020;**75**:101812. doi:10.1016/j.cpr.2019.101812

60 Clement S, Schauman O, Graham T, *et al.* What is the impact of mental health-related stigma on help-seeking? A systematic review of quantitative and qualitative studies. *Psychol Med* 2015;**45**:11–27. doi:10.1017/S0033291714000129

61 Public Health Agency of Sweden. Interventions to reduce public stigma of mental illness and suicide – are they effective? A systematic review of reviews. Stockholm: 2019.

62 Hernandez MY, Organista KC. Entertainment-Education? A Fotonovela? A New Strategy to Improve Depression Literacy and Help-Seeking Behaviors in At-Risk Immigrant Latinas. *Am J Community Psychol* 2013;**52**:224–35. doi:10.1007/s10464-013-9587-1

63 Unger JB, Cabassa LJ, Molina GB, *et al.* Evaluation of a fotonovela to increase depression knowledge and reduce stigma among hispanic adults. *J Immigr Minor Heal* 2013;**15**:398–406. doi:10.1007/s10903-012-9623-5

64 Sandelowski M. The problem of rigor in qualitative research. *ANS Adv Nurs Sci* 1986;**8**:27–37.

For peer review only

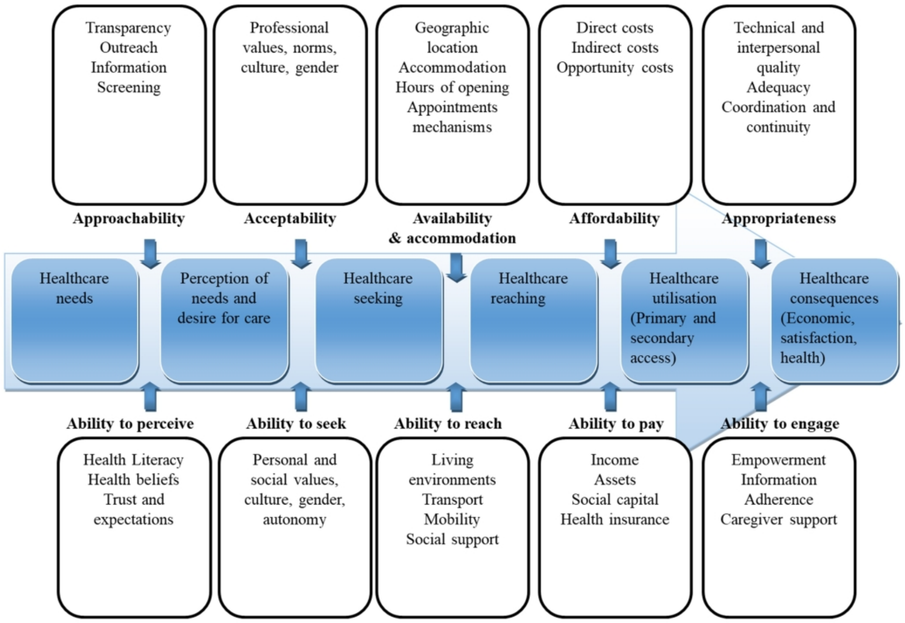

Figure 1

296x209mm (300 x 300 DPI)

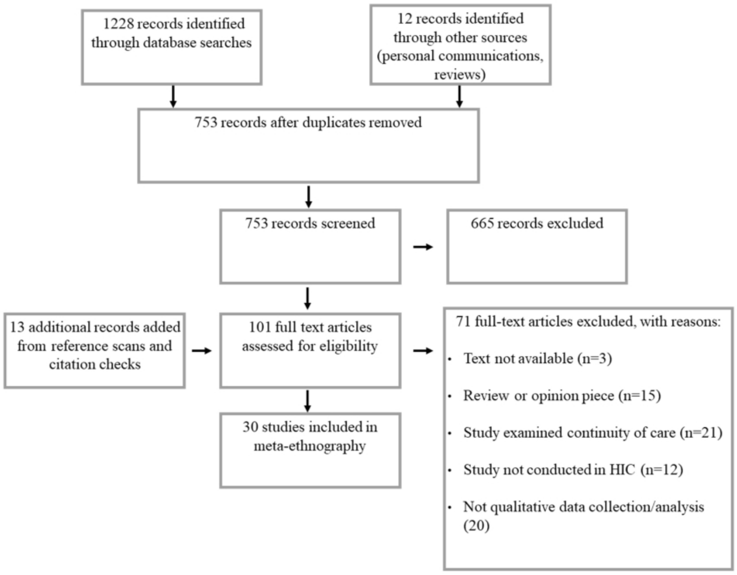

Figure 2

296x209mm (300 x 300 DPI)

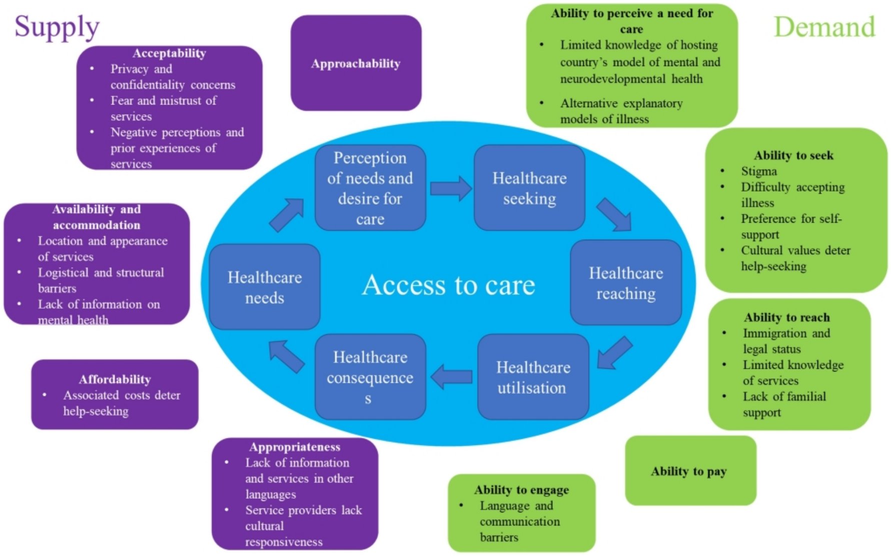

Figure 3

296x209mm (300 x 300 DPI)

exp Mental Health Services/ or Psychiatric Nursing/ or ((mental or psycholog* or psychiat*) adj3 (care or counseling or nursing or service* or treatment)).ti,ab,kf.

Culturally Competent Care/ or exp Attitude of Health Personnel/ or Health Equity/ or exp "Health Services Needs and Demand"/ or Medically Underserved Area/ or exp Health Resources/ or Delivery of Health Care/ or exp Patient Acceptance of Health Care/ or exp Attitude to Health/ or Health Services Accessibility/ or Healthcare Disparities/ or ((access* or accept* or adherence or attitude* or barrier* or compliance or deliver* or disparit* or engag* or facilitat* or hinder* or hindrance* or impede* or inequalit* or involve* or nonaccept* or non-accept or non-show* or obstacle* or participat* or refus* or seek* or usage* or utili*) adj3 (care or counseling or healthcare or health care or health-care or nursing or service* or treat*)).ti,ab,kf.

(exp Emigrants/ and Immigrants/) or Refugees/ or "Transients and Migrants"/ or "Emigration and Immigration"/ or (asylum seek* or asylum-seeker* or emigrant* or immigrant* or migrant* or refugee* or transient* or foreign born).ti,ab,kf.

(child**minor****).ti,ab,kf.

For peer review only

Supplementary Table 1. Development of third-order constructs from second-order constructs: a worked example.

Study	Second-order construct (themes from included studies)	Third-order construct (theme)
Bradby et al., 2007 [2]	Discriminatory health services	Service providers lack cultural responsiveness
Islam et al., 2017 [3]	Lack of South Asian representation in mental health care and promotion	
Posselt et al., 2017 [4]	• Lack of funding for improving the cultural responsiveness of services<br>• Services perceived as discriminatory or not culturally appropriate	
Wang et al., 2018 [5]	Providers lack cultural competence and sensitivity	
McCann et al., 2016 [6]	Perceived lack of cultural competency of formal help sources	
Choummanivong, 2013 [7]	Different understandings of an issue	
Jacobs, 2014 [8]	• Trust: lack of Somali mental health care providers<br>• Lack of provider education	
Smith, 2017 [9]	[Lack of] ethnically similar mentors	
Islam et al., 2017 [3]	Mental illness in the family	Difficulty accepting illness
de Anstiss et al., 2010 [10]	Family privacy	
Goncalves et al., 2011 [11]	• Stigma<br>• Non-recognition of the problem	
Wang et al., 2018 [5]	Denial and hiding the mental health issues	
Flink et al., 2012 [12]	Denial by daughters	
McCann et al., 2018 [13]	Personal shame deters help-seeking	
Gerdes et al., 2013 [14]	Difficulty accepting disability	
Kang-Yi et al., 2018 [15]	• Community attitudes towards autism: the many meanings of "discomfort"<br>• Discrimination and stigma	

Gill, 2010 [16]	Understanding the concept of mental health	
Zuckerman, 2014 [17]	Denial	
Smith, 2017 [9]	Unaddressed mental health concerns	

Supplementary Table 2. Third-order constructs, in the form of themes, placed within the conceptual framework of patient-centred access to healthcare. Adapted from Levesque *et al*., 2013 [1].

Determinants	Dimensions	Themes
Supply: dimensions of accessibility	Approachability	
	Acceptability	Privacy and confidentiality concerns
		Fear and mistrust of services
		Negative perceptions and prior experiences of services
	Availability & accommodation	Location and appearance of services
		Logistical and structural barriers
		Lack of information on mental health
	Affordability	Associated costs deter help-seeking
	Appropriateness	Lack of information and services in other languages
		Service providers lack cultural responsiveness
Demand: dimensions of accessibility	Ability to perceive a need for care	Limited knowledge of hosting country's model of mental and neurodevelopmental health
		Alternative explanatory models of illness
	Ability to seek	Stigma
		Difficulty accepting illness
		Preference for self-support
		Cultural values deter help-seeking
	Ability to reach	Immigration and legal status
		Limited knowledge of services
		Lack of familial support
	Ability to pay	
	Ability to engage	Language and communication barriers

REFERENCES

1 Levesque J-F, Harris M, Russell G. Patient-centred access to health care. *Int J Equity Health* 2013;**12**:1–9. doi:10.1002/cber.189502803178

2 Bradby H, Varyani M, Oglethorpe R, *et al.* British Asian families and the use of child and adolescent mental health services: A qualitative study of a hard to reach group. *Soc Sci Med* 2007;**65**:2413–24. doi:10.1016/j.socscimed.2007.07.025

3 Islam F, Multani A, Hynie M, *et al.* Mental health of South Asian youth in Peel Region, Toronto, Canada: A qualitative study of determinants, coping strategies and service access. *BMJ Open* 2017;**7**:e018265. doi:10.1136/bmjopen-2017-018265

4 Posselt M, McDonald K, Procter N, *et al.* Improving the provision of services to young people from refugee backgrounds with comorbid mental health and substance use problems: Addressing the barriers. *BMC Public Health* 2017;**17**:280. doi:10.1186/s12889-017-4186-y

5 Wang C, Do KA, Frese K, *et al.* Asian Immigrant Parents' Perception of Barriers Preventing Adolescents from Seeking School-Based Mental Health Services. *School Ment Health* 2019;**11**:364–77. doi:10.1007/s12310-018-9285-0

6 McCann T V, Mugavin J, Renzaho A, *et al.* Sub-Saharan African migrant youths' help-seeking barriers and facilitators for mental health and substance use problems: A qualitative study. *BMC Psychiatry* 2016;**16**:275. doi:10.1186/s12888-016-0984-5

7 Choummanivong C. *Refugee Youths: Adaptation and Mental Health Service Provision*. 2013.

8 Jacobs M. *Better Mental Health Service Provision for Somali Youth: Overcoming the Barriers*. 2014.

9 Smith LT. 'They might not show it and they might not say it, but in their mind they're really stressed' : a qualitative exploration of stressors and barriers to care for Latinx youth. Published Online First: 2017.https://repositories.lib.utexas.edu/bitstream/handle/2152/47450/SMITH-MASTERSREPORT-2017.pdf

10 de Anstiss H, Ziaian T. Mental health help-seeking and refugee adolescents: Qualitative findings from a mixed-methods investigation. *Aust Psychol* 2010;**45**:29–37. doi:10.1080/00050060903262387

11 Gonçalves M, Moleiro C. The family-school-primary care triangle and the access to mental health care among migrant and ethnic minorities. *J Immigr Minor Heal* 2012;**14**:682–90. doi:10.1007/s10903-011-9527-9

12 Flink IJE, Beirens TMJ, Butte D, *et al.* The role of maternal perceptions and ethnic background in the mental health help-seeking pathway of adolescent girls. *J Immigr Minor Heal* 2013;**15**:292–9. doi:10.1007/s10903-012-9621-7

13 McCann T V., Renzaho A, Mugavin J, *et al.* Stigma of mental illness and substance misuse in sub-Saharan African migrants: A qualitative study. *Int J Ment Health Nurs* 2018;**27**:956–65. doi:10.1111/inm.12401

14 Gerdes AC, Lawton KE, Haack LM, *et al.* Latino parental help seeking for childhood ADHD. *Adm Policy Ment Heal Ment Heal Serv Res* 2014;**41**:503–13. doi:10.1007/s10488-013-0487-3

15 Kang-Yi CD, Grinker RR, Beidas R, *et al.* Influence of Community-Level Cultural Beliefs about Autism on Families' and Professionals' Care for Children. *Transcult Psychiatry* 2018;**55**:623–47. doi:10.1177/1363461518779831

16 Iqbal Kaur G. *Punjabi sikh parents' beliefs about suicide and suicide-related behaviours*. 2010.

17 Zuckerman KE, Sinche B, Cobian M, *et al.* Conceptualization of Autism in the Latino

For peer review only

Community and its Relationship With Early Diagnosis. *J Dev Behav Pediatr* 2014;**35**:522–32. doi:10.1097/DBP.0000000000000091

For peer review only

BMJ Open

Perceived barriers to care for migrant children and young people with mental health problems and/or neurodevelopmental differences in high-income countries: a meta-ethnography.

Journal:	*BMJ Open*
Manuscript ID	bmjopen-2020-045923.R1
Article Type:	Original research
Date Submitted by the Author:	16-Jun-2021
Complete List of Authors:	Place, Vanessa; Karolinska Institutet, Department of Public Health Sciences<br>Nabb, Benjamin; Karolinska Institutet, Department of Public Health Sciences<br>Gubi, Ester; Karolinska Institutet, Department of Public Health Sciences<br>Assel, Karima; Karolinska Institutet, Department of Public Health Sciences<br>Åhlén, Johan; Karolinska Institutet, Department of Public Health Sciences<br>Hagström, Ana; Karolinska Institutet, Department of Public Health Sciences<br>Bäärnhielm, Sofie; Transcultural Center<br>Dalman, Christina; Karolinska Institutet, Public Health Sciences<br>Hollander, Anna-Clara; Karolinska Institutet, Department of Public Health Sciences
**Primary Subject Heading**:	Mental health
Secondary Subject Heading:	Public health, Qualitative research
Keywords:	PUBLIC HEALTH, Child & adolescent psychiatry < PSYCHIATRY, QUALITATIVE RESEARCH

SCHOLARONE™
Manuscripts

I, the Submitting Author has the right to grant and does grant on behalf of all authors of the Work (as defined in the below author licence), an exclusive licence and/or a non-exclusive licence for contributions from authors who are: i) UK Crown employees; ii) where BMJ has agreed a CC-BY licence shall apply, and/or iii) in accordance with the terms applicable for US Federal Government officers or employees acting as part of their official duties; on a worldwide, perpetual, irrevocable, royalty-free basis to BMJ Publishing Group Ltd ("BMJ") its licensees and where the relevant Journal is co-owned by BMJ to the co-owners of the Journal, to publish the Work in this journal and any other BMJ products and to exploit all rights, as set out in our licence.

The Submitting Author accepts and understands that any supply made under these terms is made by BMJ to the Submitting Author unless you are acting as an employee on behalf of your employer or a postgraduate student of an affiliated institution which is paying any applicable article publishing charge ("APC") for Open Access articles. Where the Submitting Author wishes to make the Work available on an Open Access basis (and intends to pay the relevant APC), the terms of reuse of such Open Access shall be governed by a Creative Commons licence – details of these licences and which Creative Commons licence will apply to this Work are set out in our licence referred to above.

Other than as permitted in any relevant BMJ Author's Self Archiving Policies, I confirm this Work has not been accepted for publication elsewhere, is not being considered for publication elsewhere and does not duplicate material already published. I confirm all authors consent to publication of this Work and authorise the granting of this licence.

For peer review only

Perceived barriers to care for migrant children and young people with mental health problems and/or neurodevelopmental differences in high-income countries: a meta-ethnography.

Vanessa Place[1], Benjamin Nabb[1], Ester Gubi[1], Karima Assel[1,2,], Johan Åhlen[1,3], Ana Hagström[1,4], Sofie Bäärnhielm[2, 5], Christina Dalman[1,3], Anna-Clara Hollander[1]

1. Karolinska Institutet, Department of Global Public Health, Stockholm, Sweden

2. Transkulturellt Centrum, Stockholm, Sweden

3. Centre for Epidemiology and Community Medicine, Stockholm, Sweden

4. Medical Management Centre, Department of Learning, Informatics, Management and Ethics, KI

5. Karolinska Institutet, Department of Clinical Neuroscience, Stockholm, Sweden

Corresponding author: Vanessa Place, vanessa.place@stud.ki.se, research group: Epidemiology of Psychiatric Conditions, Substance use and Social environment (EPICSS), Department of Public Health Sciences, Karolinska Institutet, Solnavägen 1E, SE-171 77, Stockholm, phone number: +46707567136

Main text word count – 7342 words

For peer review only

ABSTRACT

Objectives – To develop conceptual understanding of perceived barriers to seeking care for migrant children and young people (aged 0-25 years) with mental health problems and/or neurodevelopmental differences in high-income countries.

Design – Qualitative evidence synthesis using meta-ethnography methodology. We searched four electronic databases (Medline; PsycINFO; Global Health; and Web of Science) from inception until July 2019 for qualitative studies exploring barriers to care (as perceived by migrant communities and service providers) for migrant children and young people in high-income countries with neurodevelopmental differences and/or mental health problems. The quality of included studies was explored systematically using a quality assessment tool.

Results – We screened 753 unique citations and 101 full texts, and 30 studies met our inclusion criteria. We developed sixteen themes representing perceived barriers to care on the supply and demand side of the care-seeking process. Barriers included: stigma; fear and mistrust of services; lack of information on mental health; and service providers lacking cultural responsiveness. Themes were incorporated into Levesque and colleagues' conceptual framework of patient-centred access to healthcare, creating a version of the framework specific to migrant children and young people's mental health and neurodevelopmental differences.

Conclusions – This is the first qualitative evidence synthesis on barriers to care for mental health problems and/or neurodevelopmental differences in migrant children and young people in high-income countries. We present an adapted conceptual framework that will help professionals and policy makers to visualise the complex nature of barriers to care, and assist in improving practice and designing interventions to overcome them. Similar barriers were identified across study participants and migrant populations. While many barriers were also similar to those for children and young people in general populations, migrant families faced further, specific barriers to care. Interventions targeting multiple barriers may be required to ensure migrant families reach care.

Keywords – migrants, children and young people, mental health, neurodevelopmental disorders, care-seeking, qualitative research, meta-ethnography, qualitative evidence synthesis

ARTICLE SUMMARY

Strengths and limitations of this study

- This is the first qualitative evidence synthesis of perceived barriers to care for migrant children and young people with mental health problems and/or neurodevelopmental differences in high-income countries.
- Meta ethnography provides a systematic way of synthesising qualitative data across multiple studies, and the qualitative research synthesised here exploring the lived experiences of migrant communities can help shape future approaches to improving care access.
- The relatively large number of included studies, with diverse settings and participants, may have reduced the analytical depth of the meta-ethnography.
- The CASP (Critical Appraisal Skills Programme) quality assessment tool was used to explore the quality of included studies, but there is currently no consensus on how to assess the quality of primary qualitative studies.
- The use of meta-ethnography to modify an existing framework, instead of creating a new theory or conceptual framework, is a novel use of the methodology that can have biased author's interpretations.

For peer review only

INTRODUCTION

In 2019, the number of international migrants (people living in a country other than that in which they were born) reached 272 million, of which 33 million were children [1]. A body of evidence points to an increased risk of mental health problems amongst children and young people (aged up to 25 years) from migrant backgrounds, which does vary amongst this diverse population [2–7]. A systematic review on the mental health of refugee children in OECD countries, for example, found that they seem to experience elevated levels of psychological distress; but reported variation in the definition, measurement, and levels of distress [7], demonstrating an urgency to better understand the needs of this diverse population.

Studies have demonstrated that psychiatric care usage is lower amongst migrant groups than wider populations, an effect that reduces over time spent in the hosting country [8–11]. Studies indicate that this under-utilisation is present among migrant children and young people in HICs too [12,13]. The reasons behind this are still not understood. Thus, bringing together qualitative research exploring barriers to children and young people's mental health services is essential to form a comprehensive understanding of this phenomenon.

The initial focus of this synthesis was mental health. However, in HICs care for children with neurodevelopmental differences is often given within the mental health system, and preliminary literature searches revealed that perceived barriers were starkly similar to those for mental health problems. This demonstrated the importance of modifying the synthesis aim to include barriers to care for children with neurodevelopmental differences, including autism spectrum disorders (ASD) and attention deficit hyperactivity disorder (ADHD).

The aim of this qualitative synthesis was to develop conceptual understanding of perceived barriers to care-seeking for children and young people from a migrant background, hereafter referred to as migrant children and young people, with mental health problems and/or neurodevelopmental differences in HICs. The research question was: what are the barriers, as perceived by migrant communities and service providers, to seeking care for migrant children and young people with mental health problems and/or neurodevelopmental differences in HICs? The purpose of this synthesis was to inform the design of interventions to increase mental health care-seeking amongst newly arrived migrant children and young people in Sweden. Our chosen methodology was Nobilt and Hare's meta-ethnography (ME) [14].

The synthesis results were used to adapt Levesque and colleagues' framework of patient-centred access to healthcare [15], presented in its original form in Figure 1. This framework seeks to conceptualise dimensions of access to healthcare on the supply and demand side, allowing operationalisation of access at each stage of the care-seeking process. Care-seeking is depicted as an arrow; from need for, to consequences of, seeking care (Figure 1) [15].

The framework conceptualises five dimensions of accessibility of care, represented in the upper part of Figure 1: 1) Approachability; 2) Acceptability; 3) Availability and accommodation; 4) Affordability and 5) Appropriateness. These dimensions refer to factors on the supply side (health care facilities, systems, and providers) that can influence access to care, with examples presented in boxes (Figure 1). These dimensions of accessibility interact with five corresponding dimensions of individuals' abilities, or demand-side factors, to generate access to care. These dimensions are shown (together with examples) in the lower half of Figure 1, and include: 1) Ability to perceive; 2) Ability to seek; 3) Ability to reach; 4) Ability to pay and 5) Ability to engage.

The framework uses a multilevel perspective to consider factors related to systems, organisations, and providers, together with population, community, and individual factors [15]. The results of our meta-ethnography provide a new interpretation and application of this framework specific to migrant children and young people.

For peer review only - http://bmjopen.bmj.com/site/about/guidelines.xhtml

METHODOLOGY

We followed Nobilt and Hare's methodology for conducting a meta-ethnography, an interpretive approach to qualitative synthesis with seven steps: 1. Getting started; 2. Deciding what is relevant; 3. Reading the studies; 4. Determining how the studies are related; 5. Translating studies into each other; 6. Synthesising translations; and 7. Expressing the synthesis [14]. Meta-ethnographies are widely used in healthcare research to form higher-level interpretations, rather than simply aggregating data, so this method was suited to our aim. Reporting was structured using the eMERGe reporting guidance for meta-ethnographies [16].

Search strategy and process

Two authors (VP and BN) collaborated with information experts at Karolinska Institute University Library to develop comprehensive search strategies. Searches of 4 electronic databases (Medline; PsycINFO; Global Health; and Web of Science) were conducted from 10/06/2019 until 11/07/2019. The search strategy for Medline is presented in Supplementary Figure 1, other strategies are available upon request. Manual searches of citations on Google Scholar and reference lists were conducted to identify additional studies.

Qualitative studies exploring perceived barriers to care for migrant children and young people (aged up to 25 years) in HICs with mental health problems and/or neurodevelopmental differences were included (note that the decision to include neurodevelopmental differences was made prior to the literature searches described here). Given the range of terms for mental health problems and neurodevelopmental differences in use, and the broad scope of the synthesis, studies exploring barriers to care for mental health problems; mental disorders or illness; and neurodevelopmental differences, disorders, or conditions, were included. Neurodevelopmental differences were broadly defined based upon the definition of neurodevelopmental behavioural intellectual disorders used by the WHO, which defines these as disabilities in the functioning of the brain that affects a child's behaviour, memory, or ability to learn [17].

Studies exploring barriers to care from any perspective – children and young people, parents, or professionals – were included. Studies exploring barriers to general care-seeking, or conducted in low or middle-income countries, were beyond our scope and excluded. All years, study designs, and publication types were considered for inclusion. The search was limited to studies published in English.

Selecting primary studies

Study selection was an iterative process and search strategies were modified following new findings in the literature. Search results were imported into Mendeley reference management software for screening. Titles and abstracts were screened against the inclusion criteria by VP and BN separately, who then came together to discuss the degree of alignment in interpretation of the inclusion criteria. Through this discussion, as well as consultation with the research group, VP and BN decided which studies were eligible for full-text screening. Full-text screens were carried out in the same manner. Inter-related reliability was not formally recorded.

Data extraction and determining how studies are related

VP and BN read studies independently, paying attention to context. Next, first- and second-order constructs were extracted. Schutz [18] defined first-order constructs as participants' own interpretations, usually as direct quotes, and second-order constructs as original authors' interpretations of participants' words, typically as themes. VP and BN re-read studies, then independently extracted first- and second-order constructs from the entirety of the studies to ensure that all raw data relevant to the research question was included [19].

For peer review only

Constructs relating to barriers to care-seeking for migrant children and young people's mental health problems and neurodevelopmental differences were extracted. Given the heterogeneity of participants, conditions, and neurodevelopmental differences in included studies, it was deemed impractical to sort extracted constructs into different groups without our preconceptions influencing this process. Indeed, after much discussion, we took the view that qualitative syntheses should focus on how individuals and communities experience their health status and the care-seeking process, over arbitrary definitions and diagnoses [20].

Extracted constructs were then placed in a data extraction form in Microsoft Excel. VP and BN then discussed the extracted constructs to decide which were relevant to the synthesis question, and did not progress with the analysis until consensus was reached.

To determine how studies were related, VP and BN independently compared second-order constructs across studies, using lists that summarised these constructs to identify associations and differences. They recorded their interpretations of these constructs in a standardised table, including key concepts from each study, and then compared this information. This involved printing out the lists and tables and drawing physical arrows between concepts, in a mind-map style. An example mind-map is presented in Figure 2, showing papers that reported culturally inappropriate services (a precursor to the theme 'Service providers lack cultural responsiveness'). Interpretations and the relationships between studies were discussed within the research group in order to explore alternative interpretations and challenge preconceptions.

Finally, descriptive data was recorded in a standardised data extraction form, first piloted on three studies by VP and EG. This was used to determine how studies were related in terms of location, year, and participant group(s).

Translation and synthesis

The following steps occurred in an iterative, non-linear process, but are described stepwise for clarity. "Key" second-order constructs and their interpretations (as recorded by VP and BN) were translated into one another by VP, through comparison across all studies and grouping of key constructs to develop new interpretations in the form of themes, representing perceived barriers to care. These themes are the third-order constructs; researchers' interpretations of original authors' interpretations [21]. Discussion within the research group decided which third-order constructs were included in the synthesis.

The synthesis product, a line-of-argument synthesis [14], was developed by incorporating third-order constructs into Levesque's conceptual framework [15]. To our knowledge, there is no methodological guidance available for incorporating meta-ethnography findings into an existing framework. However, meta-ethnography does not necessarily require formation of new theory [19], so we applied the principles of incorporating primary qualitative findings into an existing framework [22], to develop a version of this framework specific to our target population and reflect critically on our own interpretations and biases. This involved sorting third-order constructs into the supply- and demand-side dimensions of the care-seeking process in the existing Levesque framework [15], an iterative process requiring continual reflection and rearrangement of constructs and the framework itself, to produce a line-of-argument synthesis. Finally, a narrative text synthesis was constructed by VP, ACH and KA, which expresses the third-order constructs in relation to the framework. Appropriate first-order constructs (quotes) were included to provide contextual richness.

Reflexivity and trustworthiness

Reflexivity involves reflecting on the role of researcher(s) and their values in the research process and knowledge construction [22]. The researchers involved in this synthesis came from a variety of countries including Sweden, Finland, and the U.K, and professional backgrounds such as healthcare

For peer review only

professionals, (nursing, psychiatry, and psychology), economics, and biochemistry. The group discussed possible preconceptions continually in order to understand how they would come to influence the interpretive process.

The trustworthiness of qualitative methodology is commonly assessed using four criteria: credibility, dependability, confirmability and transferability [23]. Credibility was achieved through the use of the eMERGe reporting guidelines [16]. Authors kept a diary throughout the process to record their reflections and held regular de-briefs to reflect on their interpretations of the data, further enhancing credibility. Dependability was attained through multiple authors independently carrying out steps of the methodology and meeting to confer. The varied backgrounds and expertise (described above) enabled authors to explore different aspects of the data, while still agreeing upon the line-of-argument synthesis, increasing the robustness of findings. Further, these varied backgrounds allowed the research team to question one another's interpretations, increasing dependability. Confirmability was enhanced by researchers acknowledging their preconceptions of the barriers to care, while reflecting on their position and preconceptions throughout the study.

An interpretive approach, focused on how people experience phenomena and the world around them [22], was the main epistemological standpoint taken during this synthesis. This was best suited to the study aim, focused on how migrant children and young people experience barriers to care.

Role of the funder

The funders of the study had no role in study design, data collection, data analysis, data interpretation, or writing of the report. The corresponding author had full access to all the data in the study and had final responsibility for the decision to submit for publication.

Patient and public involvement

No patient involved.

RESULTS

Included studies

The process of study selection is shown in Figure 3, which resulted in 30 studies being selected for inclusion [24-53].

Quality assessment

A quality assessment of included studies was carried out using an adapted version of the CASP (Critical Appraisal Skills Programme) quality assessment tool, presented in Table 1 [54]. Studies were not excluded based on these criteria, rather they were used to identify gaps in the quality of reporting [54]. Included studies were generally of good quality (Table 1), with four studies categorised as low-quality [29,39,42,46]. However, reporting of certain aspects was generally poor, such as researchers' role and justification of a qualitative approach.

Table 1. Quality criteria and results. Adapted version of the CASP quality assessment tool, taken from Atkins *et al.*, 2008.

Question	Yes	No	Unclear
Is this study qualitative research?	28	0	2
Are the research questions clearly stated?	24	6	0

For peer review only

Is the qualitative approach clearly justified?	20	7	3
Is the approach appropriate for the research question?	28	0	2
Is the study context clearly described?	27	2	1
Is the role of the researcher clearly described?	13	15	2
Is the sampling method clearly described?	27	1	2
Is the sampling strategy appropriate for the research question?	29	0	1
Is the method of data collection clearly described?	28	1	1
Is the data collection method appropriate to the research question?	26	1	3
Is the method of analysis clearly described?	25	4	1
Is the analysis appropriate for the research question?	27	2	1
Are the claims made supported by sufficient evidence?	27	1	2

Study characteristics

Detailed study characteristics are presented in Table 2. Two articles were produced from the same data set but with different research questions [36,38], thus were considered separate studies. Five included studies are theses [47–50,53]. Two studies that were not strictly classified as qualitative research (Table 1) were included as they contained relevant qualitative data; one because it included interviews with community members about use of mental health services [25], and the other [42] as it contained descriptions of qualitative data collection and analysis, if brief and incomplete.

For peer review only

Table 2. Characteristics of included studies. Note that the language used to describe participants was taken directly from included studies.

Study	Country	Type (no. and gender) participants	Participant background (no.)	Data collection method; dates	Method of analysis	Study focus
Arora *et al*., 2019 [24]	USA	Adolescents aged 14-20 years (33, 14 male and 19 female)	First- and second-generation Asian-American	FGD; dates not reported	Grounded theory approach	To obtain information on Asian-American immigrant youth's perceptions of SBMH services and recommendations to address the mental health needs of this group
Bradby *et al*., 2007 [25]	Scotland, UK	Parents (35, 5 male and 30 female), service users aged 6-14 years (7, 3 male and 4 female) and/or their parents (8, 2 male and 6 female), healthcare professionals (7, gender not stated), and carers (8, 1 male and 7 female) of children aged 5-13 years who had not been referred to CAMHS despite having suitable problems	South Asia	FGD and semi-structured interviews; September 2003 - March 2005	Thematic analysis	To explore attitudes to and experiences of CAMHS among families of South Asian origin who were underrepresented as service-users
Guzder *et al*., 2013 [26]	Canada	Parents (20, gender not stated) of children aged 7-12 years who attended a psychiatric day hospital for significant behaviour impairment	Immigrant (10) and native-born Canadian (10)	In-depth, semi-structured interviews; dates not reported	Grounded theory approach	To identify similarities and differences in perceptions of mental health problems and help seeking experiences between native-born and immigrant parents

Islam *et al*., 2017 [27]	Canada	Youth aged 15-23 years (10, 2 male and 8 female)	First- and second-generation South Asian	In-depth, semi-structured interviews; March-July 2015	Thematic analysis	To understand the mental health challenges and service access barriers experienced by South Asian youth populations
Posselt *et al*., 2017 [28]	Australia	Youth aged 12-25 years (15, 6 male and 9 female) and service providers (15, gender not stated): social workers, psychologists, and mental health nurses/nurse practitioners	Refugee youth from Afghan (9), African (4) and Bhutanese backgrounds (2); service providers background not stated	Semi-structured interviews; 2013-14	Thematic analysis	To determine the barriers and facilitators to effective, culturally responsive service provision for young people of refugee background with comorbid mental health and AOD problems
de Anstiss *et al*., 2010 [29]	Australia	Adolescents aged 13-17 years (85, 44 male and 41 female)	Refugee (various backgrounds)	FGD; dates not reported	Thematic analysis	To fill gaps in knowledge of rates and patterns of service utilisation across service sectors, use of informal supports, and actual and perceived barriers to mental health services amongst refugee adolescents
Goncalves *et al*., 2012 [30]	Portugal	Adolescents aged 12-17 (16, 11 male and 5 female), mothers (6), teachers and health professionals (17, 5 male and 12 female)	First- and second-generation immigrant adolescents and first-generation immigrant mothers (various backgrounds), teachers and health professionals background not stated	FGD; dates not reported	Content analysis	To shed light on the family-school-primary care triangle and the access to mental health care for migrant and ethnic minority families
Colucci *et al*., 2015 [31]	Australia	Service providers (115, 30 male and 85 female) from agencies that	Not stated	FGD and semi-structured interviews; April	Thematic analysis	To explore the perspectives of experienced service

		support refugee youth: mental health services, community support organisations, health services, schools, the state government health department, and a refugee resettlement agency		2010 - November 2011		providers on barriers and facilitators to engaging refugee-background young people with mental health services
Wang *et al*., 2019 [32]	USA	Adolescents (55, 10 male and 45 female) aged 11-19 years	First- and second-generation Latinx- (25) and Asian-American (27), and bi-racial (Asian and Latinx, n=3)	Semi-structured interviews; 2014-15 academic year	Descriptive thematic analysis	To explore Asian– and Latinx–American adolescents' perceptions of seeking help for mental health concerns at middle or high schools
Forrest-Bank *et al*., 2019 [33]	USA	Service providers (14, 2 male and 12 female) from organisations serving refugee youth: community non-profit organisations, public schools, healthcare agencies, local government, an advocacy group, and a church.	Caucasian (12), not stated (2)	Semi-structured interviews; dates not reported	Inductive thematic analysis	To gain the perspectives of service providers about the strengths and barriers to mental health services addressing the needs of resettled refugee youth
Flink *et al*., 2012 [34]	The Netherlands	Mothers (41) of daughters aged 10-20 years	First-generation Moroccan (13) and Turkish (17), and Dutch (11)	FGD; dates not reported	Content analysis	To examine how mothers with different ethnic backgrounds perceive the issue of help-seeking for internalising problems experienced by adolescent girls
Ellis *et al*., 2010 [35]	USA	Adolescents aged 11-20 years (30, 10 male and 20 female)	First-generation Somali or Somali-Bantu	FGD and in-depth interviews; January 2004 - June 2006	Thematic analysis	To examine the utility of the Gateway Provider Model in understanding

						service utilization and pathways to help for Somali refugee adolescents
McCann *et al*., 2018 [36]	Australia	Youth aged 16-25 years (28, 18 male and 10 female), parents and community leaders (41, 24 male and 17 female)	Sub-Saharan African youth, first-generation sub-Saharan African parents, and community leaders	FGD and semi-structured interviews; dates not reported	Inductive thematic analysis	To explore the stigma experience with mental illness and/or substance misuse amongst sub-Saharan African immigrants, and to examine the implications of this for help seeking for young people and parents from these communities
Valibhoy *et al*., 2017 [37]	Australia	Youth aged 18-25 years who had received services from a mental health professional in Australia (16, 7 male and 9 female)	Refugee (various backgrounds)	In-depth interviews; March 2012 - January 2013	Thematic analysis	To document the perspectives of youth from refugee backgrounds on their experience of accessing mental health services
McCann *et al*., 2016 [38]	Australia	Youth aged 16-25 years (28, 18 male and 10 female), parents and community leaders (41, 24 male and 17 female)	Sub-Saharan African youth, first-generation sub-Saharan African parents, and community leaders	FGD and semi-structured interviews; dates not reported	Inductive thematic analysis	To identify the help-seeking barriers and facilitators for anxiety, depression and alcohol and drug use problems in young people from recently established sub-Saharan African migrant communities
Gerdes *et al*., 2013 [39]	USA	Parents (73, 25 male and 46 female, 2 not stated) of children aged 5-12 years	Latino	Problem recognition questionnaire for ADHD; dates not reported	Grounded theory	To better understand the help seeking process that occurs within Latino families when a child is exhibiting behaviours

						consistent with ADHD
Chapman *et al*., 2014 [40]	USA	Parents (16, 1 male and 15 female) of newly immigrated youth aged 12-18 years	First-generation Latino	Semi-structured interviews; August 2004 - March 2006	Narrative analysis	To examine parental perceptions of mental health and to determine patterns of help seeking and service use
Kang-Yi *et al*., 2018 [41]	USA	Church leaders (9, 7 male and 2 female) and early childcare workers (4, 1 male and 3 female)	First-generation Korean-American	Semi-structured interviews; September 2013 - August 2014	Grounded theory approach	To understand Korean immigrant families' and professionals' beliefs and attitudes towards autism and other developmental disorders
Shor, 2007 [42]	Israel and Russia	Parents in Israel (100, 21 male and 79 female) and Russia (100, 24 male and 76 female) of children under 18 years of age	Jewish immigrant parents from the Former Soviet Union (living in Israel) and Jewish parents living in Russia	Interviews; dates not reported	Content analysis	To differentiate between culturally-based help-seeking patterns of immigrant parents and those resulting from their new social context
AlAzzam & Daack-Hirsch, 2015 [43]	USA	Mothers (16) of children aged 5-12 years	First-generation Arab Muslim	Semi-structured interviews; dates not reported	Content analysis	To elicit Arab immigrant Muslim mothers' perceptions of and responses to behavioural problems in children (especially those associated with ADHD)
Wang *et al*., 2018 [44]	USA	Parents (19, 2 male and 17 female) of children aged 12-18 years	First-generation Asian	In-depth, semi-structured interviews; dates not reported	Thematic analysis	To investigate Asian immigrant parents' perception of barriers for seeking SBMHS for their adolescents
Ling *et al*., 2014 [45]	USA	Service providers (16, 4 male and 12 female) who work with Asian-	White (3), Korean (4) and Chinese (9) ethnicity	In-depth, semi-structured interviews; March -	Consensual Qualitative Research method	To explore the perceived mental health needs of urban Asian-American

		American youth: counsellors, social workers, psychologists, organisation leaders and program coordinators, and an educator.		June 2012		adolescents and barriers to meeting their needs from the perspective of social service providers
Messent & Murrel, 2003 [46]	UK	Parents (7, 3 male and 4 female) who were attending or had attended CAMHS with their children, and social workers (4, 2 male and 2 female)	Bangladesh	Group meetings with semi-structured interview guide; dates not reported	None	To examine the accessibility of a child and adolescent mental health service to ethnic minority populations
Gill, 2010 [47]	Canada	Parents (4, 1 male and 3 female) of children aged 9-20 years, young adults aged 19-22 years (4, 1 male and 3 female), therapists and medical professionals (2 female), and a community leader (1 male)	First-generation Punjabi Sikh parents, second-generation Punjabi Sikh young adults, South-Asian mental health and medical professionals, and a Punjabi Sikh community leader	Semi-structured interviews; dates not reported	Content analysis	To describe the beliefs held by first-generation Canadian Punjabi Sikh parents about adolescent suicide and suicide-related behaviours
Choummanivong, 2013 [48]	New Zealand	Adolescents aged 13-18 years (53, 25 male and 28 female) and mental health service providers (20, 4 male and 16 female): psychologists, social workers, general practitioners, and school guidance counsellors	Refugee adolescents and health service providers from various backgrounds	FGD and semi-structured interviews; dates not reported	Thematic analysis	To provide more information about stressors impacting on refugee youth, their coping strategies, and their experience of mental health services in New Zealand
Nguyen, 2014 [49]	USA	Mothers (4) of children aged 3-12 years who had or were currently using	First-generation Vietnamese American	Semi-structured interviews; dates not reported	Content analysis (consistent with the multiple case	To describe how Vietnamese American families that have used

		mental health services			study design)	mental health services perceive and incorporate children's mental health into their lives
Jacobs, 2014 [50]	USA	Mental health and human service providers who had experience working with Somali youth (8, 1 male and 7 female)	Somali (5) and Caucasian (3)	Semi-structured interviews; dates not reported	Content analysis	To explore how human service personnel view the barriers to services faced by Somali youth and how those barriers can be overcome
Zuckerman, 2014 [51]	USA	Parents (30, 7 male and 23 female) of typically developing children aged 2-10 years	First- and second-generation Latino	FGD and semi-structured interviews; dates not reported	Thematic analysis	To assess the understanding and conceptualisation of ASD in the Latino community in order to understand potential community barriers to early diagnosis
Araujo *et al*., 2017 [52]	USA	Caregivers (13, 1 male and 12 female) of children aged 7-10 years with ADHD symptoms that were receiving the Collaborative Life Skills program in school	Latino	Semi-structured interviews; dates not reported	Thematic analysis	To explore emotional, social, and cultural experiences of Latino youth with ADHD symptoms and their families
Smith, 2017 [53]	USA	Community members that provide services within communities with elevated rates of at-risk Latinx youth (11, 2 male and 1 female)	United States (9), Mexico (1) and Germany (1)	Qualitative interviews; December 2014 - March 2016	Grounded theory	To explore stressors and barriers to care in the Latinx youth community through a community-based participatory research framework

ADHD – attention deficit hyperactivity disorder; ASD – autism spectrum disorders; AOD – alcohol and other drugs; CAMHS – Child and Adolescent Mental Health Services; FGD – focus group discussions; SBMHS – school-based mental health services; Latinx – person(s) of Latin American origin or descent.

For peer review only

Study synthesis

Sixteen, third-order constructs, in the form of themes representing barriers to care, were developed from first- and second-order constructs extracted from included studies. A worked example of the synthesis process is presented in Supplementary Table 1, demonstrating how third-order constructs were generated from the second-order constructs. These third-order constructs were then incorporated into Levesque's conceptual framework of access to healthcare [15]. This involved categorising constructs into the dimensions of accessibility and ability that influence the care-seeking process on the supply and demand side of care-seeking, respectively, presented in the original framework [15] (Figure 1). Our third-order constructs, categorised into these dimensions, are presented in Table 3.

The adapted framework is presented in Figure 4. It employs the supply- and demand-side (dimensions of accessibility and ability, respectively) organisation of barriers to care from the original framework (Figure 1) but has been modified to include our third-order constructs in order to create a model specific to our target population. In addition, the circular shape of the adapted framework differs from the linear representation of care-seeking in the original framework, in order to encapsulate the dynamic nature of care-seeking and barriers to care, as experienced by study participants (Figure 4). Similarly, barriers to care were not assigned to a particular step in the care-seeking process, as in the original framework, to better reflect our findings (Figure 1).

Third-order constructs, within their respective dimensions of abilities and accessibility of the care-seeking process, are described below. First-order constructs (direct quotes) are presented to illustrate these constructs.

Of note, the term "participants" indicates that a concept was discussed across groups of participants in included studies (Table 2). Concepts that were mentioned specifically by, or about, a particular group are specified as such. The same system is employed for neurodevelopmental differences and mental health problems; where a specific condition was in focus, this is indicated in the text. Disconfirming cases were present both within and between studies and were included in the synthesis. For example, some study participants described seeking help for their mental health problems within their family, while others stated that they would avoid seeking help from anyone, including family, in order to avoid burdening them.

Table 3. Third-order constructs, in the form of themes, incorporated into a conceptual framework of patient-centred access to healthcare. Adapted from Levesque *et al.*, 2013.

Determinants	Dimensions	Third-order constructs
Supply: dimensions of accessibility	Approachability	
	Acceptability	Privacy and confidentiality concerns
		Fear and mistrust of services
		Negative perceptions and prior experiences of services
	Availability & accommodation	Logistical and structural barriers
		Lack of information on mental health
	Affordability	Associated costs deter help-seeking
	Appropriateness	Lack of information and services in other languages

For peer review

		Service providers lack cultural responsiveness
Demand: dimensions of ability	Ability to perceive a need for care	Alternative explanatory models
	Ability to seek	Stigma
		Preference for self-support
		Cultural values deter help-seeking
	Ability to reach	Immigration and legal status
		Limited knowledge of services
		Lack of familial support
	Ability to pay	
	Ability to engage	Language and communication barriers

Supply - dimensions of accessibility

Acceptability

Privacy and confidentiality concerns

Privacy and confidentiality concerns were identified as a significant barrier to seeking care for children's neurodevelopmental differences and mental health problems in seven included studies. Participants explained that in migrant communities problems were often kept "in-house" [37] to protect the family from the heavy stigma attached to mental health problems. One woman stated that in her community, "people will do nothing, only gossip" [25] in the face of such problems. The consequences of disclosure were perceived to outweigh any benefits of care-seeking.

Similarly, young people expressed privacy concerns about school-based mental health services; they feared that information would be shared with teachers, peers, or parents, affecting their academic grades and social life. Parents worried that care-seeking would appear on the child's transcript and negatively impact their future. Some students, however, felt that school-based mental health services provided more privacy as they were detached from their community.

The fear of providers particularly interpreters, breaching confidentiality was repeatedly identified as a barrier to care-seeking for mental health problems, as one young person explained:

"If I knew that person, and they knew my family, I wouldn't go near that place cos they might tell our parents." [29].

Fear and mistrust of services

Closely linked to privacy concerns was a fear and mistrust of services, identified in ten included studies. Parents explained that health and school systems were unfamiliar and mistrusted, reducing the likelihood they would seek help there. Young people echoed this notion for school-based mental health services and emphasised a general mistrust of adults and professionals as unable or unwilling to help.

Fear of authority and providing personal information were identified as significant barriers to mental health care, particularly for refugee families. Service providers explained that these families were often fearful of the care-seeking process:

"For people who have been through certain traumas and come from countries with very difficult political situations, providing that amount of information on paper, in black and white on the referral form can be really confronting..." [31]

In addition, participants described a fear of retribution, such as deportation or involvement of social services. Another service provider explained:

"Lack of trust also is a big problem…they think if I tell them [service providers] this will they tell the police? Will they tell the child protection agencies?" [28]

Negative perceptions and prior experiences of services

Perceptions and prior experiences of services were considered critical when deciding to seek care for children and young people in seven included studies. Negative past experiences of, and beliefs about, care-seeking spread through migrant communities, reducing the acceptability of seeking care. Furthermore, participants reported that mental health services were perceived as overly reliant on medication, culturally unacceptable, and discriminatory. One young person stated:

"If you are born in Australia you get more respect- they [health services] care more about you because you are part of them, one of them. Rather than coming from overseas you get treated... [trails off]." [28]

Young people described school-based mental health service providers as unable to relate to their concerns and not "real" [32] mental health professionals, which formed a barrier to care-seeking. Adults within migrant communities explained that mental health services were perceived as illegitimate and ineffective, giving advice that family or friends could provide, or that service providers did not take them seriously. Seeking care was therefore considered a waste of time.

Availability and Accommodation

Logistical and structural barriers

Participants in twelve included studies identified logistical and structural barriers that migrant families faced to seeking care for children's neurodevelopmental differences and mental health problems. Complex healthcare systems meant that families often found the agencies and professionals involved confusing. Furthermore, complex referral processes, underfunded services, and long waiting times led to youth disengaging with or not seeking care. Appointment-based systems, restrictions on the number and length of appointments, and inflexible opening hours were also reported as barriers to care for these conditions.

Some mental health service providers also expressed frustration over strict eligibility criteria that excluded migrant young people who may benefit from services, as one provider recalled:

"I have found people over 25 who would fit into our youth program, and fit in very well, but because of the age limit, I can't offer them that, even though I think it would probably benefit them hugely." [31]

Service providers in particular identified the location and appearance of services as a barrier to care-seeking for migrant young people. They described existing services as lacking the privacy needed due to heavy stigmatisation of mental illness. One professional recalled:

For peer review only

"…a client had to go to [a service] and when she walked out of the building, other people were actually waiting at a bus stop which was right in front and they said to her: "Why do you go in that place? That's for crazy people."" [31]

Moreover, some providers described their facilities as too clinical or sterile, and inappropriate for migrant young people. The low appeal and stigmatisation of services was perceived as an obstacle to care-seeking.

Lack of information on mental health

Participants in five included studies identified lack of information on mental health as a barrier to seeking care for children and young people's mental health problems. They reported that little or no information was available within migrant communities on the symptoms of mental illness and importance of mental health. If information was available, it was often not delivered in a way that was considered meaningful by communities. Moreover, one youth participant described how the school curriculum focused solely on physical health:

"They only teach it or recognize physical unhealthy things in your body. Like if you are feeling sick or you feel nauseous or tummy is not feeling well…but there is never anything about if you feel like crying, if you feel very sad, if you feel very angry all the time." [27]

This lack of information was believed to contribute to a lack of openness to seeking care.

Affordability

Associated costs deter help-seeking

The financial costs associated with accessing services were identified as a barrier to seeking care in five included studies, with participants highlighting that migrant families often lacked the time, insurance coverage, and money required. Some participants stated that mental health problems take "years to treat" [44], incurring costs that families could not afford. Service providers highlighted loss of income as a key deterrent:

"Refugee families are working really hard you know, and income is absolutely essential for them… and if we're going to be calling them away from their work schedule, that's a loss of income for them in order to attend these appointments." [48]

Participants in certain settings identified the cost of treatment as a barrier, explaining that it was not always affordable for migrant families. Others, conversely, highlighted that perceived cost deterred care-seeking. They explained that in some countries, specialist care (for mental health or neurodevelopmental differences) was a luxury few could afford. Even if this was not the case in their new homeland, the misconception stopped families from seeking care.

The stressors associated with poverty were identified as a further barrier to care-seeking by providers; they reported that, when money was short, young people could feel pressured to drop out of school to support the family. This was perceived to isolate young people from community resources, reducing the likelihood that they would seek help.

Appropriateness

Lack of information and services in other languages

Language and communication issues were identified as a barrier to migrant families seeking and engaging with services in nine included studies. Participants from migrant communities reported that information was often not available in their mother tongue or was poor quality, while service providers stated that a lack of information for those with a low literacy level stopped them from accessing services.

For peer review only

A shortage of bilingual service providers was perceived as a barrier in various studies, as this left families reliant on interpreters. Providers emphasised that the availability of interpreters varied, with some services only available by phone, or that the quality was low. This was perceived to damage family-provider relationships and discourage further care-seeking within communities. Indeed, not all providers were willing to use interpreters. One mental health service provider expressed their frustration:

"Most of our interpreters they… don't have enough experience. So, when people become proficient they leave for another job and leave this to a beginner. And the beginners they cannot make the things effective. So sometimes they mislead the people more often than are helpful." [33]

Interpreting services for newer migrant communities were reported to be of poorer quality, forming a further barrier to care for newly arrived families.

Service providers lack cultural responsiveness

Lack of cross-cultural responsiveness among service providers was perceived to limit the value of help-seeking for children and young people with neurodevelopmental differences or mental health problems, in eight included studies. In contrast to concerns over confidentiality, participants from migrant communities expressed a preference for same-culture service providers, who could understand their values and experiences. These participants emphasised that service providers were lacking in knowledge of the communities they serve, while providers themselves expressed frustration at their insecurities and lack of insight when working with migrant communities: "I'm walking into this almost blind." [50] Moreover, providers lacking cross-cultural awareness were viewed as judgemental of migrant families. One mother shared an anecdote from her community:

"…the counsellor started asking how you [a girl] were treated when you were young, some routine questions. Then halfway through, the counsellor was shocked by some of the difficult ways or how the parenting practices [are] normal…He basically thought this girl was abused, emotionally…so the girl, instead of getting help with her mental illness, she started to defend her parents." [44]

Demand - dimensions of ability

Ability to perceive a need for care

Alternative explanatory models

Lack of exposure to the 'Western' models of mental health and neurodevelopmental differences was identified as a barrier to seeking care within migrant communities in twelve included studies. Participants expressed a understanding of the concept of mental health that differed from how it is understood in their new homeland. This included that mental health problems do not affect children; are lifelong; and are only present within certain communities. One young person stated:

"I've never met an African suffering from anxiety. Depression is not very common in my community." [36]

Alternative explanatory models for mental health and for neurodevelopmental differences were reported to impede professional care-seeking within migrant communities. Mental illness was described by some migrant participants as contagious; while others believed that it was due to a curse or possession by an evil spirit, thus care would be sought from witchdoctors or spiritual leaders. Another prevalent idea was that neurodevelopmental differences reflected the family's faults, so care-seeking was avoided to protect the family's standing within the community. One church leader explained:

"Koreans – even if they're living in the US – tend to think that a child's disorder is the result of someone wrongdoings, or something bad that runs in the family." [41]

For peer review only

In addition, participants reported that these mental health problems were attributed to poor family relations, children who were "spoiled" [44] or lacked discipline. Some members of the migrant community stated that mental illness was due to over-protection of young people in Western societies.

Aside from different cultural understanding, a general lack of awareness of mental health within migrant communities was also reported. Participants attributed this to various causes, including that mental health was "taboo" [30], and that these conditions were difficult to conceptualise. Some parents described feeling bewildered at the onset of their children's mental illness, which they had not initially recognised as illness, while others described difficulty distinguishing between normal and abnormal behaviour.

Ability to seek

Stigma

The stigma attached to neurodevelopmental differences and mental health problems in migrant communities was highlighted as a barrier to seeking care across participant groups in twenty nine of the included studies. Some migrant community members stated that seeking help from trusted, informal sources would minimise the risk of gossip and community rejection. However, stigma was also cited as a barrier to community members (such as childcare workers) suggesting that a family seek help for a child with neurodevelopmental differences, which could be interpreted as an insult.

Participants reported that mental illness and neurodevelopmental differences could even damage a child's marriage prospects and the family's reputation. The cost of seeking care was therefore deemed too high:

"The parents have to find a daughter-in-law for themselves. That's the real point. Like no one, no one, will accept the proposal of crazy guy." [29]

This stigma extended to care-seeking, which was described as a sign of weakness and was associated with stereotypes including being "psycho" [29]. Moreover, some young people stated that their parents' reluctance to seek care for their own mental health problems had perpetuated their internalised stigma towards help-seeking.

Participants reported that the stigma attached to neurodevelopmental differences and mental health problems made them difficult to accept and could lead to denial.

"[Vietnamese parents] Don't want to look and admit that their children are sick [e.g. suffering with mental illness]. Their children need help and will not admit it. They say that their children are beautiful, just like that." [49]

Preference for self-support

Both adults and young people identified a preference for self-support as a barrier to seeking mental health care in seven included studies. One's problems were viewed as a burden that should not be placed on others; a view that was particularly prevalent amongst young people but could also stop parents from seeking support from family or health professionals.

According to some parents, young people from migrant backgrounds preferred self-support as they want to feel more independent. Young people themselves reasoned that everyone experienced these types of problems, thus they should be able to cope alone. One participant stated:

"I didn't wanna tell anyone 'cause I didn't think I had a problem, and I could fix it on my own. So, I feel like a lot of kids feel like that…That they can just fix it on their own." [32]

For peer review only

In addition, participants reported that young people feared being coerced into seeking care if they discussed their problems with their parents. This fear of losing control formed a barrier to problem-sharing and care-seeking.

Cultural values deter help-seeking

In nineteen included studies, certain cultural values within migrant communities were perceived as a barrier to help-seeking for neurodevelopmental differences and mental health problems. Participants from migrant communities expressed a preference for informal support, such as friends or churches, explaining that those who knew a child with a neurodevelopmental difference were better equipped to help. Professional help was often described as a last resort.

Moreover, some participants identified prioritising academic success over mental health as a barrier to care-seeking. Students from Asian communities reported that a fear of not living up to the 'model minority' stereotype could deter their peers from seeking help for mental health problems. Other perceived barriers included a culture of not complaining, and low priority placed on mental health and individual needs. Latinx (person(s) of Latin American origin or descent) participants emphasised how central *familismo* (the importance of the family) is in their culture. Young people were expected to prioritise the family's needs over their own; one young person stated:

"You don't talk about emotions, you don't deal with mental health, it doesn't exist. You just need to get a job, go to school, do what you need to do, pay your bills, buy your house." [27]

Ability to reach

Immigration and legal status

Concerns over immigration and legal status were identified as important barriers to care within migrant communities, including amongst those residing legally, in four included studies. Participants explained that (particularly undocumented) migrants feared that seeking mental health care would lead to deportation, while some incorrectly believed that refugees were not entitled to these services. This led some parents to minimise contact with government organisations, impeding care-seeking for their children. One participant explained:

"Going to family doctor, as an African migrant, we are scared to put somebody in the system, like if it is at early stage of mental illness, because everything here is documented. If you are on the system, it will be very hard to get rid of it." [36]

Limited knowledge of services

Limited knowledge of the mental health care system made it difficult for migrant families to understand how to seek care for their children in nine included studies. One young person noted, "Even if they were kind of aware of it [services], they wouldn't know where to start." [47]

Participants described common misconceptions surrounding who could seek mental health services. Young people, for example, incorrectly stated that school-based mental health services were only for severe mental illness or academic concerns. Similarly, participants emphasised that knowledge of the types of healthcare providers was often low within migrant communities, thus families were unsure where to seek help. Contrastingly, second-generation migrants were often better-informed and more likely to seek care than newly arrived migrants.

Lack of familial support

Lack of familial support and communication were identified as important barriers to care-seeking in six included studies. Adolescents described how discussing their mental health with their parents resulted in dismissive responses, which deterred further communication. Some explained that their

For peer review only

parents did not understand, while others said that parents were more concerned about their academic grades.

Similarly, participants reported about communication barriers within migrant families. Disagreeing with parents was not always culturally acceptable, which prevented open discussions around mental health. In addition, some young people said that their parents handled their emotions in an unhealthy way and treated help-seeking with disdain. One participant described her father's struggles to express his feelings:

"I feel like my dad, is just like…traditional thinking of Pakistani men. Right? Unfortunately. Like it has nothing to do with oh, I will miss you or anything. [i.e. he does not express how much he loves his children.]" [27]

Ability to engage

Language and communication barriers

Limited language proficiency was identified as a barrier to families engaging with services in eleven included studies, Participants highlighted that parents with poor language skills lacked the confidence to communicate with schools and service providers, preventing them from benefitting from resources. Although young people generally were considered to have fewer difficulties, this was not always the case - some young people reported struggling to express concepts related to mental health in their new language. This formed a communication barrier between families and providers, but also between parents and children with different levels of language proficiency. One participant from a migrant community explained:

"We don't really have words [in Somali], like a word for depression or anxiety, but we have words like someone being stressed out, worrying a lot…but those are just temporary states though, it's not like over a long period of time." [50]

DISCUSSION

Summary of findings

This qualitative synthesis produced sixteen third-order constructs in the form of themes, representing perceived barriers to seeking care for children and young people's neurodevelopmental differences and/or mental health problems within migrant communities in HICs. These themes were incorporated into Levesque's conceptual framework of patient-centred access to healthcare [15], creating a version of this framework specific to our target population (Figure 4). The third-order constructs demonstrate that migrant children and young people experience a range of barriers to care on the supply and demand sides of the care-seeking process. Many of these barriers are similar to those faced by children and young people in general populations, but our results demonstrate that migrant communities face additional, specific barriers to care.

To our knowledge, this is the first qualitative synthesis on perceived barriers to care for children and young peoples' neurodevelopmental differences and mental health problems within migrant communities in HICs. The adapted framework provides a snapshot of the perceived barriers to seeking care from the perspective of those who experience them, while highlighting that these barriers exist and interact within the context of communities and systems (Figure 4). Given the heterogeneity of settings and migrant groups included in the synthesis, we envision that the framework can be used as a tool (alongside in-depth knowledge of local contexts) to inform policy and service provision on migrant children and young people's mental and neurodevelopmental health. We invite researchers to comment on and expand the framework to improve its utility within this field.

For peer review only

Most included studies were conducted in the USA (n=15) or Australia (n=6); unsurprising given that both were in the top 20 destinations for all migrants in 2019 [55]. However, a lack of studies from other countries such as the UK (n=1) was unexpected and could indicate a lack of research despite high numbers of resident migrants [55]. The number of included studies, particularly for neurodevelopmental differences (n=5), was also lower than expected, which could indicate a need for more research on how migrant communities experience and perceive barriers to children and young people's neurodevelopmental and mental health care.

The fact that 22 studies focused on perceived barriers within specific migrant group(s) may reflect a move away from a "one size fits all" approach to migrant health. However, the key finding of the synthesis is that the barriers to care were repeatedly identified across study participants (parents, young people, service providers, and different migrant communities), on the supply and demand side of care seeking (Figure 4). A recent systematic review found that refugees, asylum seekers, and undocumented migrants in Europe faced common (and very similar) barriers to care, including lack of knowledge of healthcare systems and trust in health professionals [2], supporting our findings. There is currently no systematic review or meta-analysis on barriers to mental health care for migrant children and young people, however, which could further strengthen our findings.

Furthermore, many of the barriers to seeking care have also been identified for children and young people in general populations [56]. A recent systematic review of qualitative and quantitative studies on barriers to mental health care amongst children and adolescents (which included two studies in this synthesis [29,30]) reported many similar barriers, including: lack of knowledge of mental health and mental health services, and preference for self-reliance [57]. This suggests that these barriers are associated with children and young people's care-seeking, or care-seeking for stigmatised conditions, such as mental health [58]. Indeed, several barriers identified in this synthesis, such as stigma or lack of familial support, have been linked to the stigmatisation of mental health amongst children and young people in HICs [57].

Our findings suggest that migrant families often perceive multiple barriers to care, several of which are likely specific to migrant communities or ethnic minorities, including: language and communication barriers, immigration and legal status, and lack of cultural responsiveness among providers (Figure 4). These additional barriers to seeking care for migrant children and young people may contribute to low care-seeking amongst this group [2,13], and interventions targeting multiple barriers may be needed to engage migrant families.

Participants in included studies expressed frustration at the lack of bicultural providers and cultural responsiveness among service providers. However, interpreters from one's own community were considered likely to spread gossip, highlighting how interactions between barriers to care present challenges to overcoming them. Cultural sensitivity training for providers is one option to improve relationships with migrant communities and increase care-seeking, but the evidence for this is limited [59], and alone is unlikely to address the diversity of cultures and practices within different migrant communities.

Stigmatisation of mental health and neurodevelopmental differences formed a common thread through the results of this synthesis, connecting barriers such as fear and mistrust of services, and confidentiality concerns. The stigma associated with mental health has been associated with reduced care-seeking within migrant populations [11, 60]. Our findings support the notion that stigma exists at many levels, fostering misunderstanding and mistrust of services and professionals [59]. Tackling stigma is complex, likely requiring long-term interventions to improve health literacy, alter attitudes and behaviour. A recent systematic review of reviews on interventions to reduce stigma related to mental health and suicide in general populations found that some interventions were successful at improving knowledge and attitudes in the short-term, but there was a lack of evidence on interventions for stigma surrounding less severe mental health problems [61]. Although studies have attempted to

For peer review only

tackle stigma to increase mental health care-seeking within migrant communities [62,63], more research on how to effectively reduce this stigma amongst migrant children and young people is needed.

Limitations and methodological considerations

This study has several limitations. The relatively large number of included studies (n=30), with diverse settings and participants, may have reduced the analytical depth of the meta-ethnography. Translating subgroups of studies according to design may have improved the depth of the analysis, but was not considered feasible given the sheer variety of designs included. Moreover, given that similar barriers were identified across studies, the effect is judged to be minimal.

Using the meta-ethnography methodology in a novel way, to adapt an existing framework, may have limited the methodological viability of this process and may have biased author's interpretations of findings towards the existing framework's dimensions. The quality assessment of included studies was not used to exclude studies, reducing the trustworthiness of findings. Our search strategy was limited to studies published in English, which may partly explain the high number of studies from English-speaking countries (n=27), a limitation of this study. Moreover, although a broad research aim and comprehensive search strategy were employed, it was not possible to search all relevant databases and journals, thus relevant studies may have been missed.

As literature searches were performed in 2019, additional literature searches of the MedLINE databases were performed on 03/06/2021. This search produced several articles eligible for inclusion in the review [64–66], and their findings supported our findings on barriers to care. One qualitative study of facilitators and barriers for engagement in mental health treatment amongst Latino youth, for example, reported logistical barriers, beliefs about depression, and negative experiences of treatment as barriers [64].

Finally, a meta-analysis of quantitative data was not conducted, which could provide evidence on how barriers to care affect care-seeking and allow comparison between studies on perceived and actual barriers to care. However, increasing our understanding of how migrant families perceive barriers to care through a qualitative synthesis is a critical first step to overcoming these challenges.

Implications for practice and future research

The similarities in perceived barriers to care for mental health and/or neurodevelopmental differences between children and young people in general and migrant populations suggests that broader public health interventions, such as increased mental health education in schools or public awareness campaigns, could increase care-seeking across the groups. However, our results demonstrate that migrant communities perceive further, specific barriers to care, which must be carefully mapped to ensure that interventions are tailored as required.

Our results demonstrate a need to increase trust in mental health services within migrant communities, and improving communication around neurodevelopmental differences and mental health could reduce the stigma associated with help-seeking. Increasing numbers of bicultural practitioners and improving cultural responsiveness, alongside these interventions, will be critical to increase trust and ensure community engagement.

This meta-ethnography highlights several gaps in the field. While our findings suggest that perceived barriers are broadly similar between children and young people in general populations and migrant communities, this needs to be confirmed via meta-analysis. This could identify any differences between these groups in perceived barriers and service access, to better inform policy and intervention design. In addition, more studies exploring how migrant communities perceive and experience barriers to care is essential. Finally, research on interventions that facilitate care-seeking for migrant children and young people's mental health problems and neurodevelopmental differences are much needed.

For peer review only

This should involve input from migrant community members, to ensure that interventions are well-suited to the communities they seek to reach.

Conclusion

This meta-ethnography synthesised the findings of 30 qualitative studies examining perceived barriers to seeking care for migrant children and young people with neurodevelopmental differences and/or mental health problems in HICs. Its findings demonstrate that care-seeking is a dynamic process involving many interacting barriers, of which migrant families face additional, specific barriers to care. This presents a challenge to designing effective policy and interventions, but we envision that our adapted conceptual framework can serve as a tool to help professionals and policy makers visualise barriers within the wider system context.

Much further research is needed to dissect how migrant status affects the barriers to care faced by children and young people and to design effective interventions to overcome these barriers and ensure that families reach services.

FIGURE LEGENDS

Figure 1. A conceptual framework of patient-centred access to healthcare. Adapted from Levesque *et al.*, 2013 [15].

Figure 2. Determining how studies are related to one another using mind-mapping: a worked example.

Figure 3. Study selection. HIC – high-income country.

Figure 4. A conceptual framework of patient-centred access to mental health care for migrant children and young people. Adapted from Levesque *et al.*, 2013 [15].

Supplementary Figure 1. Search strategy for Medline.

Contributor statement

CD and ACH conceived the project. VP and BN carried out the database searches, screened titles, abstracts, and full texts, and extracted data together with EG. VP conducted the synthesis and wrote the manuscript. ACH provided supervision to VP and critically revised the manuscript at all stages. ACH, CD, JÅ, KA, AH and SB reviewed the study findings and read and approved the final version before submission.

Competing interests

None declared.

Funding

This work was supported by Swedish research council (Vetenskapsrådet) grant number: 2018-05763.

ACH was funded by Forte grant number: 2016-00870.

Data sharing statement

Not applicable.

Ethics statement

All data included in this article is secondary data, already published with ethical approval hence no additional permission is needed.

REFERENCES

For peer review only

1 United Nations Department of Economic and Social Affairs Population Division. International Migrant Stock 2019. 2019. https://www.un.org/en/development/desa/population/migration/data/estimates2/countryprofiles.asp

2 Priebe S, Giacco D, El-Nagib R. Public health aspects of mental health among migrants and refugees : a review of the evidence on mental health in the WHO European Region. Copenhagen: 2016.

3 Fazel M, Reed R V., Panter-Brick C, *et al.* Mental health of displaced and refugee children resettled in high-income countries: Risk and protective factors. *Lancet* 2012;**379**:266–82. doi:10.1016/S0140-6736(11)60051-2

4 Kim Y, Evans BE, Hagquist C. Mental Health Problems Among Adolescents in Sweden from 1995 to 2011: The Role of Immigrant Status and the Proportions of Immigrant Adolescents in Their Surrounding Community. *J Immigr Minor Heal* 2020;**22**:232–9. doi:10.1007/s10903-019-00951-0

5 Vervliet M, Meyer Demott MA, Jakobsen M, *et al.* The mental health of unaccompanied refugee minors on arrival in the host country. *Scand J Psychol* 2014;**55**:33–7. doi:10.1111/sjop.12094

6 Lindström M, Rosvall M, Modén B. Country of birth, parental background and self-rated health among adolescents: A population-based study. *Scand J Public Health* 2014;**42**:743–50. doi:10.1177/1403494814545104

7 Bronstein I, Montgomery P. Psychological Distress in Refugee Children: A Systematic Review. *Clin Child Fam Psychol Rev* 2011;**14**:44–56. doi:10.1007/s10567-010-0081-0

8 Manhica H, Almquist Y, Rostila M, *et al.* The use of psychiatric services by young adults who came to Sweden as teenage refugees: A national cohort study. *Epidemiol Psychiatr Sci* 2017;**26**:526–34. doi:10.1017/S2045796016000445

9 Jablonska B, Kosidou K, Ponce de Leon A, *et al.* Neighborhood Socioeconomic Characteristics and Utilization of ADHD Medication in Schoolchildren: A Population Multilevel Study in Stockholm County. *J Atten Disord* 2020;**24**:265–76. doi:10.1177/1087054716643257

10 Brendler-Lindqvist M, Norredam M, Hjern A. Duration of residence and psychotropic drug use in recently settled refugees in Sweden - a register-based study. *Int J Equity Health* 2014;**13**.http://www.equityhealthj.com/content/pdf/s12939-014-0122-2.pdf

11 Satinsky E, Fuhr DC, Woodward A, *et al.* Mental health care utilisation and access among refugees and asylum seekers in Europe: A systematic review. *Health Policy (New York)*

For peer review only

2019;**123**:851–63. doi:10.1016/j.healthpol.2019.02.007

12 Colucci. The utilisation of mental health services by children and young people from a refugee background: A systematic literature review. Int. J. Cult. Ment. Health. 2014;**7**:86.

13 Markkula N, Cabieses B, Lehti V, *et al.* Use of health services among international migrant children - a systematic review. *Global Health* 2018;**14**:52. doi:10.1186/s12992-018-0370-9

14 Nobilt G, Hare R. *Meta-ethnography: synthesizing qualitative studies*. California: : SAGE Publications Inc 1988.

15 Levesque J-F, Harris M, Russell G. Patient-centred access to health care. *Int J Equity Health* 2013;**12**:1–9. doi:10.1002/cber.189502803178

16 France EF, Cunningham M, Ring N, *et al.* Improving reporting of meta-ethnography: The eMERGe reporting guidance. *BMC Med Res Methodol* 2019;**19**:1–13. doi:10.1186/s12874-018-0600-0

17 Calamandrei G, Friza H, van den Hazel P, *et al.* Baseline Report on Neurodevelopmental disorders in the framework of the European Environment and Health Strategy. 2003. http://coombs.anu.edu.au/SpecialProj/ASAA/biennial-conference/2006/Ramzan-Yuko-ASAA2006.pdf

18 Schutz A. *Collected Papers I: The Problem of Social Reality*. 1962.

19 Toye F, Seers K, Allcock N, *et al.* Meta-ethnography 25 years on: Challenges and insights for synthesising a large number of qualitative studies. *BMC Med Res Methodol* 2014;**14**:1–14. doi:10.1186/1471-2288-14-80

20 Campbell R, Pound P, Pope C, *et al.* Evaluating meta-ethnography: A synthesis of qualitative research on lay experiences of diabetes and diabetes care. *Soc Sci Med* 2003;**56**:671–84. doi:10.1016/S0277-9536(02)00064-3

21 Britten N, Campbell R, Pope C, *et al.* Using meta ethnography to synthesise qualitative research: A worked example. *J Heal Serv Res Policy* 2002;**7**:209–15. doi:10.1258/135581902320432732

22 Green J, Thorogood N. *Qualitative Methods for Health Research*. 4th ed. London: : SAGE Publications Ltd STM 2018.

23 Sandelowski M. The problem of rigor in qualitative research. *ANS Adv Nurs Sci* 1986;**8**:27–37.

24 Arora PG, Algios A. School-Based Mental Health for Asian American Immigrant Youth: Perceptions and Recommendations. *Asian Am J Psychol* 2018;**10**:166–81.

For peer review only

doi:10.1037/aap0000142

25 Bradby H, Varyani M, Oglethorpe R, *et al.* British Asian families and the use of child and adolescent mental health services: A qualitative study of a hard to reach group. *Soc Sci Med* 2007;**65**:2413–24. doi:10.1016/j.socscimed.2007.07.025

26 Guzder J, Yohannes S, Zelkowitz P. Helpseeking of immigrant and native born parents: A qualitative study from a montreal child day hospital. *J Can Acad Child Adolesc Psychiatry* 2013;**22**:275–81.http://ovidsp.ovid.com/ovidweb.cgi?T=JS&PAGE=reference&D=prem1&NEWS=N&AN=24223046

27 Islam F, Multani A, Hynie M, *et al.* Mental health of South Asian youth in Peel Region, Toronto, Canada: A qualitative study of determinants, coping strategies and service access. *BMJ Open* 2017;**7**:e018265. doi:10.1136/bmjopen-2017-018265

28 Posselt M, McDonald K, Procter N, *et al.* Improving the provision of services to young people from refugee backgrounds with comorbid mental health and substance use problems: Addressing the barriers. *BMC Public Health* 2017;**17**:280. doi:10.1186/s12889-017-4186-y

29 de Anstiss H, Ziaian T. Mental health help-seeking and refugee adolescents: Qualitative findings from a mixed-methods investigation. *Aust Psychol* 2010;**45**:29–37. doi:10.1080/00050060903262387

30 Gonçalves M, Moleiro C. The family-school-primary care triangle and the access to mental health care among migrant and ethnic minorities. *J Immigr Minor Heal* 2012;**14**:682–90. doi:10.1007/s10903-011-9527-9

31 Colucci E, Minas H, Szwarc J, *et al.* In or out? Barriers and facilitators to refugee-background young people accessing mental health services. *Transcult Psychiatry* 2015;**52**:766–90. doi:10.1177/1363461515571624

32 Wang C, Barlis J, Do KA, *et al.* Barriers to Mental Health Help Seeking at School for Asian– and Latinx–American Adolescents. *School Ment Health* Published Online First: 2019. doi:10.1007/s12310-019-09344-y

33 Forrest-Bank SS, Held ML, Jones A. Provider Perspectives of Services Addressing the Mental Health Needs of Resettled Refugee Youth. *Child Adolesc Soc Work J* Published Online First: 2019. doi:10.1007/s10560-019-00602-1

34 Flink IJE, Beirens TMJ, Butte D, *et al.* The role of maternal perceptions and ethnic background in the mental health help-seeking pathway of adolescent girls. *J Immigr Minor Heal* 2013;**15**:292–9. doi:10.1007/s10903-012-9621-7

For peer review only

35 Ellis BH, Lincoln AK, Charney ME, *et al.* Mental Health Service Utilization of Somali Adolescents: ReligioN, Community, and School as Gateways to Healing. *Transcult Psychiatry* 2010;**47**:789–811. doi:10.1177/1363461510379933

36 McCann T V., Renzaho A, Mugavin J, *et al.* Stigma of mental illness and substance misuse in sub-Saharan African migrants: A qualitative study. *Int J Ment Health Nurs* 2018;**27**:956–65. doi:10.1111/inm.12401

37 Valibhoy MC, Kaplan I, Szwarc J. 'it comes down to just how human someone can be': A qualitative study with young people from refugee backgrounds about their experiences of Australian mental health services. *Transcult Psychiatry* 2017;**54**:23–45. doi:10.1177/1363461516662810

38 McCann T V, Mugavin J, Renzaho A, *et al.* Sub-Saharan African migrant youths' help-seeking barriers and facilitators for mental health and substance use problems: A qualitative study. *BMC Psychiatry* 2016;**16**:275. doi:10.1186/s12888-016-0984-5

39 Gerdes AC, Lawton KE, Haack LM, *et al.* Latino parental help seeking for childhood ADHD. *Adm Policy Ment Heal Ment Heal Serv Res* 2014;**41**:503–13. doi:10.1007/s10488-013-0487-3

40 Chapman M V., Stein GL. How do new immigrant Latino parents interpret problem behavior in adolescents? *Qual Soc Work* 2014;**13**:270–87. doi:10.1177/1473325012468478

41 Kang-Yi CD, Grinker RR, Beidas R, *et al.* Influence of Community-Level Cultural Beliefs about Autism on Families' and Professionals' Care for Children. *Transcult Psychiatry* 2018;**55**:623–47. doi:10.1177/1363461518779831

42 Shor R. Differentiating the culturally-based help-seeking patterns of immigrant parents from the former Soviet Union by comparison with parents in Russia. *Am J Orthopsychiatry* 2007;**77**:216–20. doi:10.1037/0002-9432.77.2.216

43 AlAzzam M, Daack-Hirsch S. Arab Immigrant Muslim Mothers' Perceptions of Children's Attention Deficit Hyperactivity Disorder. *Procedia - Soc Behav Sci* 2015;**185**:23–34. doi:10.1016/j.sbspro.2015.03.454

44 Wang C, Do KA, Frese K, *et al.* Asian Immigrant Parents' Perception of Barriers Preventing Adolescents from Seeking School-Based Mental Health Services. *School Ment Health* 2019;**11**:364–77. doi:10.1007/s12310-018-9285-0

45 Ling A, Okazaki S, Tu MC, *et al.* Challenges in Meeting the Mental Health Needs of Urban Asian American Adolescents: Service Providers' Perspectives. *Race Soc Probl* 2014;**6**:25–37. doi:10.1007/s12552-014-9117-2

For peer review only

46 Messent P, Murrell M. Research Leading to Action: A Study of Accessibility of a CAMH Service to Ethnic Minority Families. *Child Adolesc Ment Health* 2003;**8**:118–24. doi:10.1111/1475-3588.00057

47 Iqbal Kaur G. *Punjabi sikh parents' beliefs about suicide and suicide-related behaviours*. 2010.

48 Choummanivong C. *Refugee Youths: Adaptation and Mental Health Service Provision*. 2013.

49 Nguyen NH. *Vietnamese American Families' Perceptions of Children's Mental Health: How Culture Impacts Utilization of Mental Health Services*. 2014.

50 Jacobs M. *Better Mental Health Service Provision for Somali Youth: Overcoming the Barriers*. 2014.

51 Zuckerman KE, Sinche B, Cobian M, *et al.* Conceptualization of Autism in the Latino Community and its Relationship With Early Diagnosis. *J Dev Behav Pediatr* 2014;**35**:522–32. doi:10.1097/DBP.0000000000000091

52 Araujo EA, Pfiffner L, Haack LM. Emotional, Social and Cultural Experiences of Latino Children with ADHD Symptoms and their Families. *J Child Fam Stud* 2017;**26**:3512–24. doi:10.1007/s10826-017-0842-1

53 Smith LT. 'They might not show it and they might not say it, but in their mind they're really stressed' : a qualitative exploration of stressors and barriers to care for Latinx youth. Published Online First: 2017.https://repositories.lib.utexas.edu/bitstream/handle/2152/47450/SMITH-MASTERSREPORT-2017.pdf

54 Atkins S, Lewin S, Smith H, *et al.* Conducting a meta-ethnography of qualitative literature: Lessons learnt. *BMC Med Res Methodol* 2008;**8**:1–10. doi:10.1186/1471-2288-8-21

55 International Organisation for Migration. World Migration Report 2020. Geneva: 2020. doi:10.1002/wom3.2

56 Gulliver A, Griffiths KM, Christensen H. Perceived barriers and facilitators to mental health help-seeking in young people: a systematic review. *BMC Psychiatry* 2010;**10**:113. doi:10.1186/1471-244X-10-113

57 Radez J, Reardon T, Creswell C, *et al.* Why do children and adolescents (not) seek and access professional help for their mental health problems? A systematic review of quantitative and qualitative studies. *Eur Child Adolesc Psychiatry* Published Online First: 2020. doi:10.1007/s00787-019-01469-4

58 Corrigan PW, Watson AC. Understanding the impact of stigma on people with mental illness. *World Psychiatry* 2002;**1**:16–20.https://pubmed.ncbi.nlm.nih.gov/16946807

For peer review only

59 Clement S, Schauman O, Graham T, *et al.* What is the impact of mental health-related stigma on help-seeking? A systematic review of quantitative and qualitative studies. *Psychol Med* 2015;**45**:11–27. doi:10.1017/S0033291714000129

60 Byrow Y, Pajak R, Specker P, *et al.* Perceptions of mental health and perceived barriers to mental health help-seeking amongst refugees: A systematic review. *Clin Psychol Rev* 2020;**75**:101812. doi:10.1016/j.cpr.2019.101812

61 Public Health Agency of Sweden. Interventions to reduce public stigma of mental illness and suicide – are they effective? A systematic review of reviews. Stockholm: 2019.

62 Hernandez MY, Organista KC. Entertainment-Education? A Fotonovela? A New Strategy to Improve Depression Literacy and Help-Seeking Behaviors in At-Risk Immigrant Latinas. *Am J Community Psychol* 2013;**52**:224–35. doi:10.1007/s10464-013-9587-1

63 Unger JB, Cabassa LJ, Molina GB, *et al.* Evaluation of a fotonovela to increase depression knowledge and reduce stigma among hispanic adults. *J Immigr Minor Heal* 2013;**15**:398–406. doi:10.1007/s10903-012-9623-5

64 Stafford AMC, Draucker CB. Barriers to and Facilitators of Mental Health Treatment Engagement Among Latina Adolescents. *Community Ment Health J* 2020;**56**:662–9. doi:10.1007/s10597-019-00527-0

65 Baak M, Miller E, Ziersch A, *et al.* The Role of Schools in Identifying and Referring Refugee Background Young People Who Are Experiencing Mental Health Issues. *J Sch Health* 2020;**90**:172–81. doi:10.1111/josh.12862

66 Chiang SY, Fenaughty J, Lucassen MFG, *et al.* Navigating double marginalisation: migrant Chinese sexual and gender minority young people's views on mental health challenges and supports. *Cult Heal Sex* 2019;**21**:807–21. doi:10.1080/13691058.2018.1519118

For peer review only

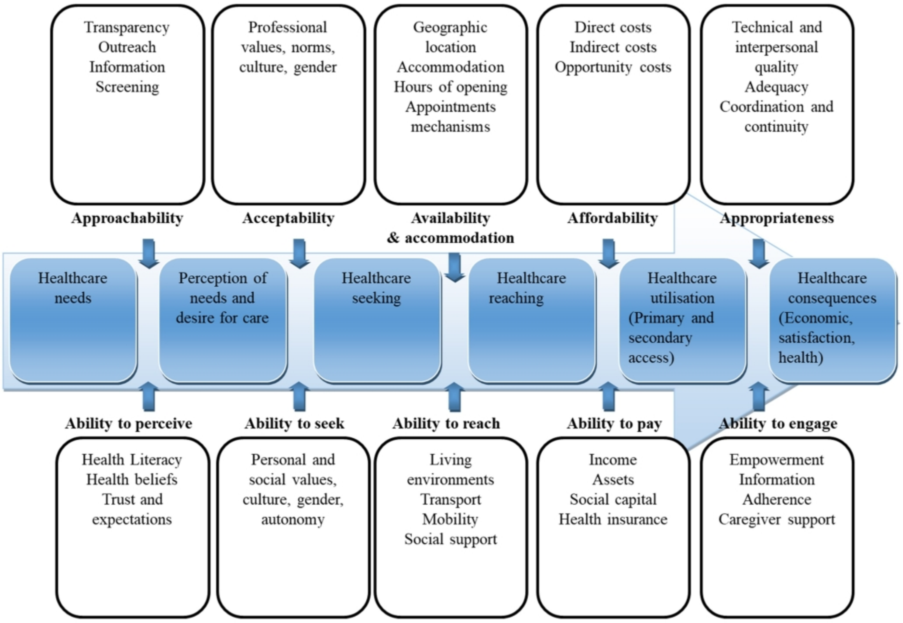

Figure 1

296x209mm (300 x 300 DPI)

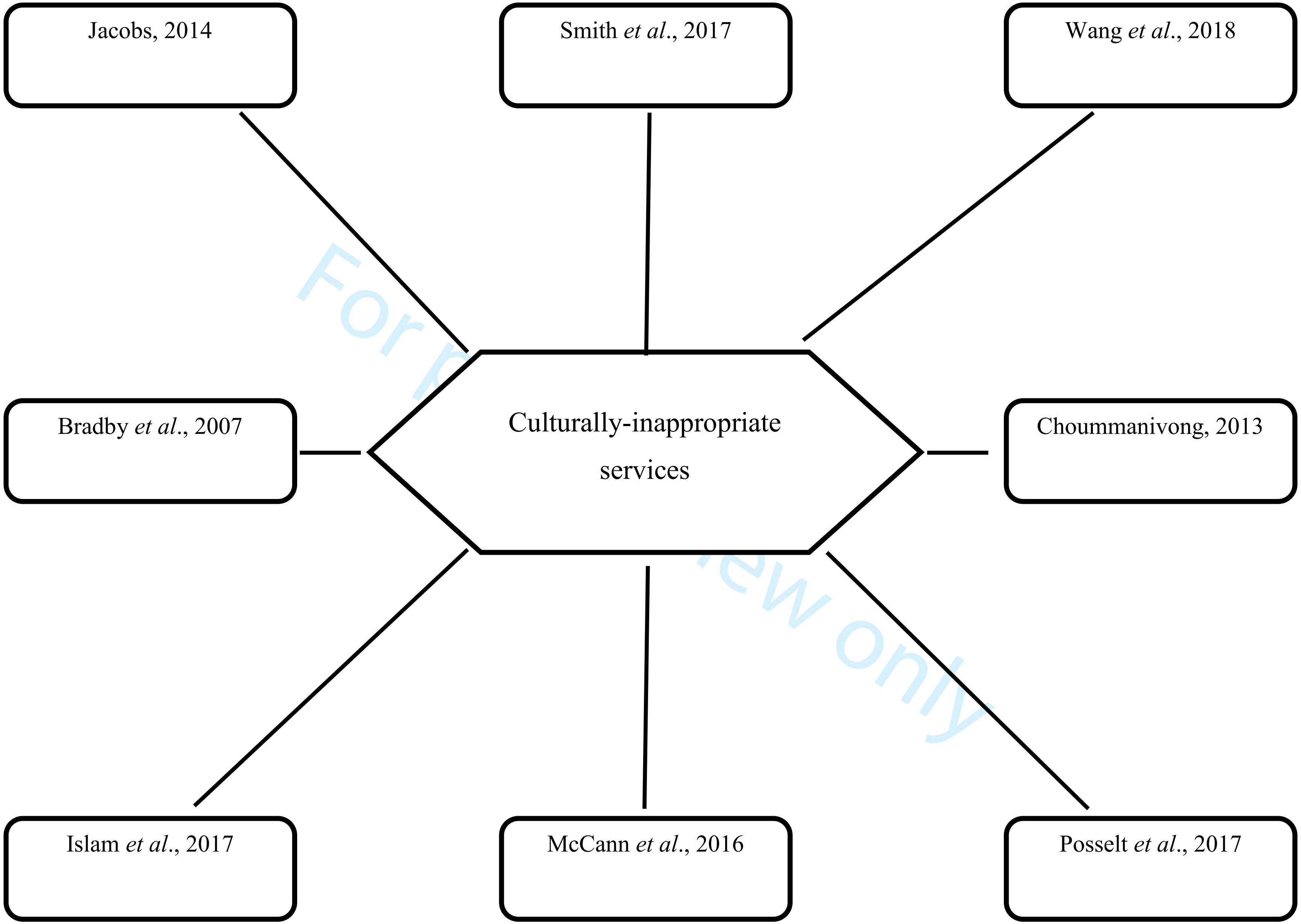
Jacobs, 2014
Smith *et al*., 2017
Wang *et al*., 2018
Bradby *et al*., 2007
Culturally-inappropriate services
Choummanivong, 2013
Islam *et al*., 2017
McCann *et al*., 2016
Posselt *et al*., 2017

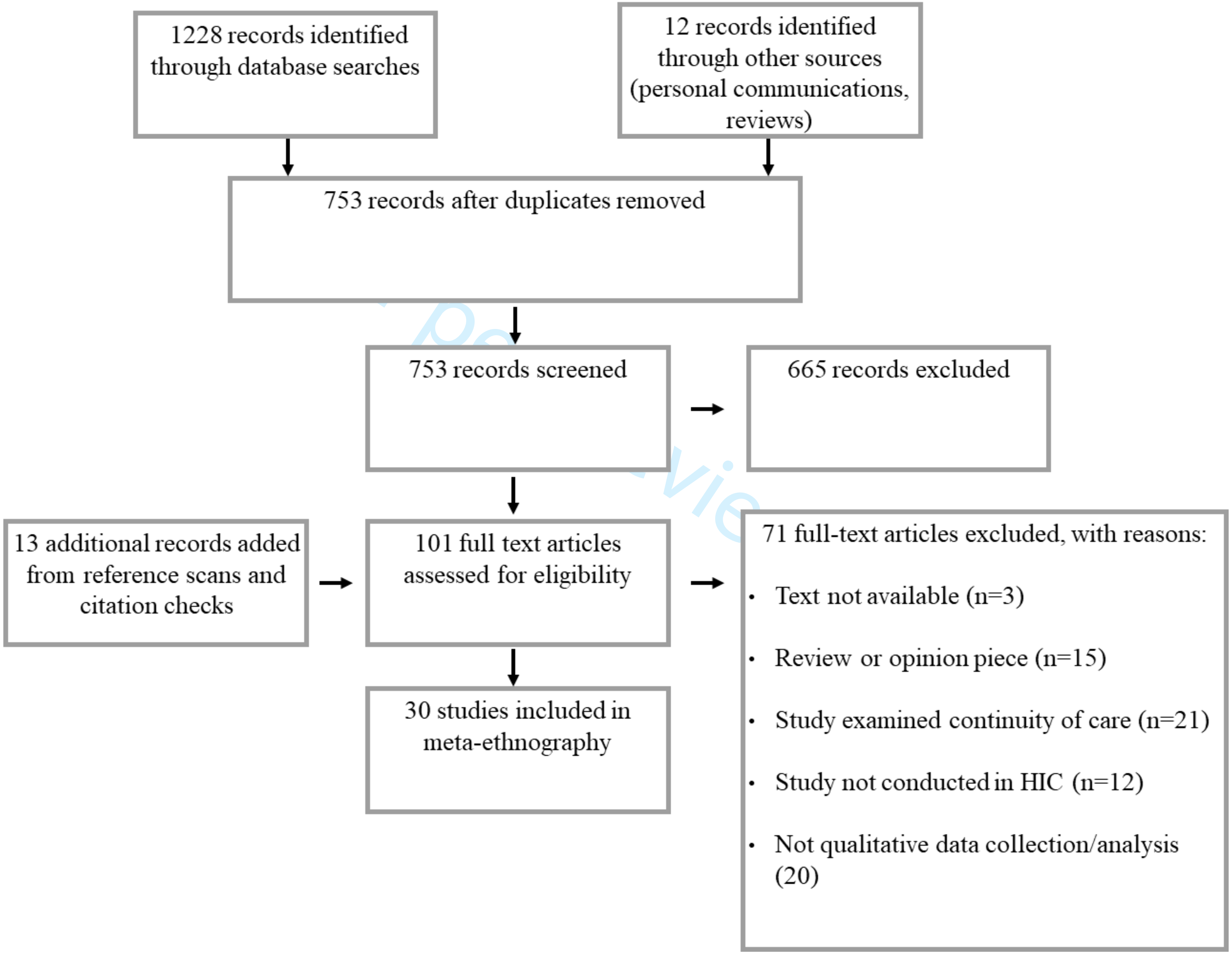
1228 records identified through database searches
12 records identified through other sources (personal communications, reviews)
753 records after duplicates removed
753 records screened
665 records excluded
13 additional records added from reference scans and citation checks
101 full text articles assessed for eligibility
71 full-text articles excluded, with reasons:
• Text not available (n=3)
• Review or opinion piece (n=15)
• Study examined continuity of care (n=21)
• Study not conducted in HIC (n=12)
• Not qualitative data collection/analysis (20)
30 studies included in meta-ethnography

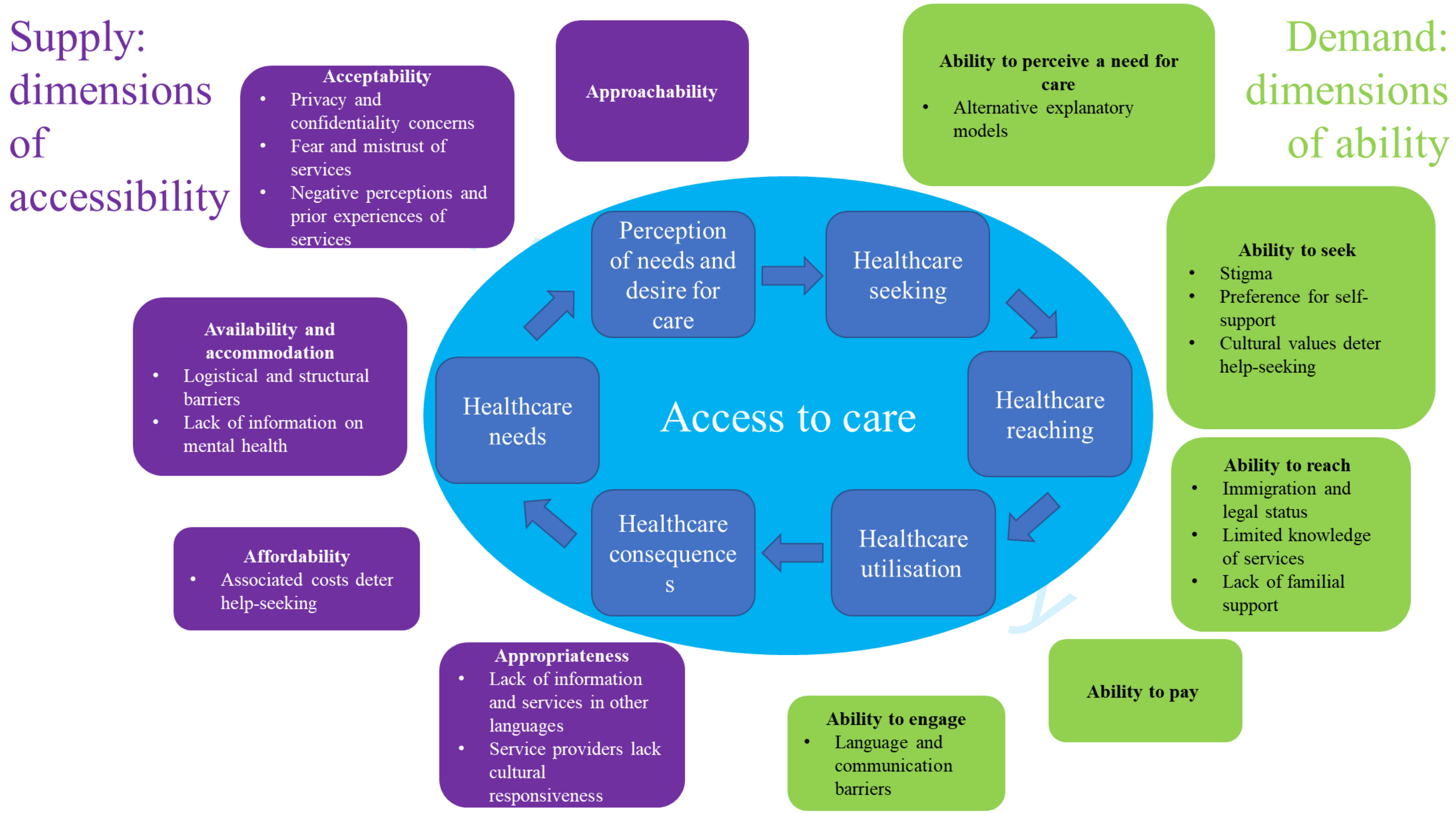
Supply: dimensions of accessibility
Acceptability
• Privacy and confidentiality concerns
• Fear and mistrust of services
• Negative perceptions and prior experiences of services
Approachability
Ability to perceive a need for care
• Alternative explanatory models
Demand: dimensions of ability
Perception of needs and desire for care
Healthcare seeking
Ability to seek
• Stigma
• Preference for self-support
• Cultural values deter help-seeking
Availability and accommodation
• Logistical and structural barriers
• Lack of information on mental health
Healthcare needs
Access to care
Healthcare reaching
Ability to reach
• Immigration and legal status
• Limited knowledge of services
• Lack of familial support
Affordability
• Associated costs deter help-seeking
Healthcare consequences
Healthcare utilisation
Ability to pay
Appropriateness
• Lack of information and services in other languages
• Service providers lack cultural responsiveness
Ability to engage
• Language and communication barriers

exp Mental Health Services/ or Psychiatric Nursing/ or ((mental or psycholog* or psychiat*) adj3 (care or counseling or nursing or service* or treatment)).ti,ab,kf.

Culturally Competent Care/ or exp Attitude of Health Personnel/ or Health Equity/ or exp "Health Services Needs and Demand"/ or Medically Underserved Area/ or exp Health Resources/ or Delivery of Health Care/ or exp Patient Acceptance of Health Care/ or exp Attitude to Health/ or Health Services Accessibility/ or Healthcare Disparities/ or ((access* or accept* or adherence or attitude* or barrier* or compliance or deliver* or disparit* or engag* or facilitat* or hinder* or hindrance* or impede* or inequalit* or involve* or nonaccept* or non-accept or non-show* or obstacle* or participat* or refus* or seek* or usage* or utili*) adj3 (care or counseling or healthcare or health care or health-care or nursing or service* or treat*)).ti,ab,kf.

(exp Emigrants/ and Immigrants/) or Refugees/ or "Transients and Migrants"/ or "Emigration and Immigration"/ or (asylum seek* or asylum-seeker* or emigrant* or immigrant* or migrant* or refugee* or transient* or foreign born).ti,ab,kf.

(child**minor****).ti,ab,kf.

For peer review only

Supplementary Table 1. Development of third-order constructs from second-order constructs: a worked example.

Study	Second-order construct (themes from included studies)	Third-order construct (new theme)
Bradby et al., 2007 [1]	Discriminatory health services	Service providers lack cultural responsiveness
Islam et al., 2017 [2]	Lack of South Asian representation in mental health care and promotion	
Posselt et al., 2017 [3]	• Lack of funding for improving the cultural responsiveness of services<br>• Services perceived as discriminatory or not culturally appropriate	
Wang et al., 2018 [4]	Providers lack cultural competence and sensitivity	
McCann et al., 2016 [5]	Perceived lack of cultural competency of formal help sources	
Choummanivong, 2013 [6]	Different understandings of an issue	
Jacobs, 2014 [7]	• Trust: lack of Somali mental health care providers<br>• Lack of provider education	
Smith, 2017 [8]	[Lack of] ethnically similar mentors	

REFERENCES

1 Bradby H, Varyani M, Oglethorpe R, *et al.* British Asian families and the use of child and adolescent mental health services: A qualitative study of a hard to reach group. *Soc Sci Med* 2007;**65**:2413–24. doi:10.1016/j.socscimed.2007.07.025

2 Islam F, Multani A, Hynie M, *et al.* Mental health of South Asian youth in Peel Region, Toronto, Canada: A qualitative study of determinants, coping strategies and service access. *BMJ Open* 2017;**7**:e018265. doi:10.1136/bmjopen-2017-018265

3 Posselt M, McDonald K, Procter N, *et al.* Improving the provision of services to young people from refugee backgrounds with comorbid mental health and substance use problems: Addressing the barriers. *BMC Public Health* 2017;**17**:280. doi:10.1186/s12889-017-4186-y

4 Wang C, Do KA, Frese K, *et al.* Asian Immigrant Parents' Perception of Barriers Preventing Adolescents from Seeking School-Based Mental Health Services. *School Ment Health* 2019;**11**:364–77. doi:10.1007/s12310-018-9285-0

5 McCann T V, Mugavin J, Renzaho A, *et al.* Sub-Saharan African migrant youths' help-seeking barriers and facilitators for mental health and substance use problems: A qualitative study. *BMC Psychiatry* 2016;**16**:275. doi:10.1186/s12888-016-0984-5

6 Choummanivong C. *Refugee Youths: Adaptation and Mental Health Service Provision*. 2013.

For peer review only - http://bmjopen.bmj.com/site/about/guidelines.xhtml

7 Jacobs M. *Better Mental Health Service Provision for Somali Youth: Overcoming the Barriers*. 2014.

8 Smith LT. ‘They might not show it and they might not say it, but in their mind they’re really stressed’ : a qualitative exploration of stressors and barriers to care for Latinx youth. Published Online First: 2017.https://repositories.lib.utexas.edu/bitstream/handle/2152/47450/SMITH-MASTERSREPORT-2017.pdf

For peer review only
